# Tor1/Sch9-Regulated Carbon Source Substitution Is as Effective as Calorie Restriction in Life Span Extension

**DOI:** 10.1371/journal.pgen.1000467

**Published:** 2009-05-08

**Authors:** Min Wei, Paola Fabrizio, Federica Madia, Jia Hu, Huanying Ge, Lei M. Li, Valter D. Longo

**Affiliations:** 1 Andrus Gerontology Center, University of Southern California, Los Angeles, California, United States of America; 2 Department of Biological Sciences, University of Southern California, Los Angeles, California, United States of America; 3 Department of Molecular and Computational Biology, University of Southern California, Los Angeles, California, United States of America; Stanford University Medical Center, United States of America

## Abstract

The effect of calorie restriction (CR) on life span extension, demonstrated in organisms ranging from yeast to mice, may involve the down-regulation of pathways, including Tor, Akt, and Ras. Here, we present data suggesting that yeast Tor1 and Sch9 (a homolog of the mammalian kinases Akt and S6K) is a central component of a network that controls a common set of genes implicated in a metabolic switch from the TCA cycle and respiration to glycolysis and glycerol biosynthesis. During chronological survival, mutants lacking *SCH9* depleted extracellular ethanol and reduced stored lipids, but synthesized and released glycerol. Deletion of the glycerol biosynthesis genes *GPD1*, *GPD2*, or *RHR2*, among the most up-regulated in long-lived *sch9*Δ, *tor1*Δ, and *ras2*Δ mutants, was sufficient to reverse chronological life span extension in *sch9*Δ mutants, suggesting that glycerol production, in addition to the regulation of stress resistance systems, optimizes life span extension. Glycerol, unlike glucose or ethanol, did not adversely affect the life span extension induced by calorie restriction or starvation, suggesting that carbon source substitution may represent an alternative to calorie restriction as a strategy to delay aging.

## Author Summary

Studies using model organisms have pointed to the existence of evolutionarily conserved genes and signaling pathways that regulates life span. Changes in the activity of these genes/pathways have also been implicated in mediating the beneficial effect of calorie restriction, a well-recognized intervention that extends the life span from yeast to mammals. We investigated the global gene expression changes and identified genes involved in the metabolism of various kinds of carbon sources that are associated with longevity in the single cell organism, the baker's yeast. Although glucose and ethanol are common carbon sources for growth, they also have detrimental pro-aging effects in yeast. Long-lived yeast mutants actively utilize available glucose and ethanol and produce glycerol, which does not adversely affect the yeast life span extension. Our finding suggest that this “carbon source substitution” observed in long-lived yeast creates an environment mimicking calorie restriction, which together with the direct regulation of stress resistance systems optimizes life span extension. Findings using these simple genetic models will help to elucidate fundamental longevity regulatory mechanisms and identify similar pathways in mammals.

## Introduction

Mutations that decrease the activities of the Akt/PKB, Tor, and Ras pathways extend the lifespan of several model organisms, suggesting that the underlying mechanisms of longevity regulation are conserved in many eukaryotic organisms [1],[2]. Akt/PKB is a highly conserved serine-threonine kinase shown to function in the Daf-2 longevity pathway of *Caenorhabditis elegans* [3]. Homologous longevity modulating pathways were also identified in *Drosophila* and mice [2]. In yeast, Sch9, which shares high sequence identity with the mammalian kinases Akt/PKB and S6K, is part of a nutrient-sensing pathway whose downregulation extends the chronological lifespan (CLS, the survival time of a population of non-dividing yeast) by up to 2-fold [4]. The Ras G-proteins are also evolutionary conserved and implicated in cell division in response to glucose/growth factors. The deletion of *RAS2* doubles the CLS of yeast [5]. In mammals, a role for Ras in longevity control has not been established conclusively but, together with Akt, Ras is one of the major mediators of IGF-I signaling, which has been shown to promote aging [6],[7]. Another conserved nutrient-responsive pathway, regulating cell growth and cell-cycle progression, involves the protein kinase target of rapamycin, TOR, which has been associated with life span regulation in *C. elegans* and *Drosophila*. Knockdown of LET-363/CeTOR, starting at the first day of the adult life, more than doubled the life span of worm [8]. Similarly, a reduced activity of Daf-15, the worm ortholog of the mammalian mTOR-interacting protein raptor, promotes life span extension [9]. In flies, overexpression of dominant-negative dTOR or TOR-inhibitory dTsc1/2 proteins also leads to longevity extension [10]. Moreover, knockdown of CeTOR does not further extend the life span of worms subject to dietary restriction (DR) and inhibition of TOR protects flies from the deleterious effects of rich food, suggesting the beneficial effect of DR is, at least in part, mediated by TOR [10],[11].

Two TOR orthologs, *TOR1* and *TOR2*, have been identified in yeast. Both Tor1 and Tor2 mediate growth-related signaling in a rapamycin-sensitive manner, whereas Tor2 has an additional rapamycin-insensitive function in controlling the cell-cycle-dependent organization of actin cytoskeleton [12]. Reduction of the TOR complex I (TORC1) activity results in an extension of yeast replicative life span (RLS), the number of daughter cells generated by individual mother cells [13],^14^, comparable to that obtained when Sch9 is inactivated [15],[16]. Furthermore, a high throughput assay to measure the CLS of individual yeast deletion mutants identified several long-lived strains carrying deletions of genes implicated in the Tor pathway [17]. Additional evidence supporting an inverse correlation between Tor1 activity and CLS has recently been provided [18].

The aging-regulatory function of both yeast Tor1 and Sch9 mediates the calorie restriction (CR)-dependent RLS extension. The down-regulation of either pathway mimics the effect of lowering the glucose content of the medium, and no further extension of RLS is observed when the *sch9*Δ or the *tor1*Δ mutants are calorie restricted [19]. Ethanol produced during fermentative growth is used as carbon source during diauxic shift and post-diauxic phase, when the yeast cells switch from rapid growth to slow budding and eventually cease proliferation [20],[21]. Switching yeast grown in glucose/ethanol medium to water models an extreme CR/starvation condition for non-dividing cells. This severe form of CR doubles chronological survival of wild type yeast [22]. In contrast to RLS, CR-induced increase of CLS is only partially mediated by Sch9 [23],[24].

Despite the extensive body of work demonstrating a link between nutrient-sensing pathways and life span regulation in different organisms, the key mechanisms responsible for delaying the aging process are still elusive. The direct correlation between life span extension and the ability to withstand different stress challenges, which has been observed in different model organisms, indicates that the activation of cellular protection represents an important survival strategy [25]. Our previous studies suggest that superoxide plays an important role in aging and age-dependent mortality, but protection against superoxide only accounts for a small portion of the potent effect of mutations in *SCH9* and *RAS2* on life span [5]. The connection between calorie restriction and the Sch9, Tor and Ras2 pathways as well as the mechanisms of CR-dependent effects on life span remain poorly understood. Here we present evidence that changes in the expression of a set of genes controlled by Sch9 as well as Tor and Ras lead to a metabolic switch to glycerol production, which, together with the direct regulation of stress resistance systems, causes enhanced cellular protection and life span extension. Unlike the pro-aging carbon sources glucose and ethanol, glycerol does not elicit adverse effect on calorie restriction-induced cellular protection and life span extension suggesting that Tor1/Sch9-regulated glycerol biosynthesis leads to a carbon source substitution (CSS) that is as effective as calorie restriction in life span extension.

## Results

### Genetic Interactions between *SCH9*, and *RAS2* and *TOR1*

**Figure 1:**
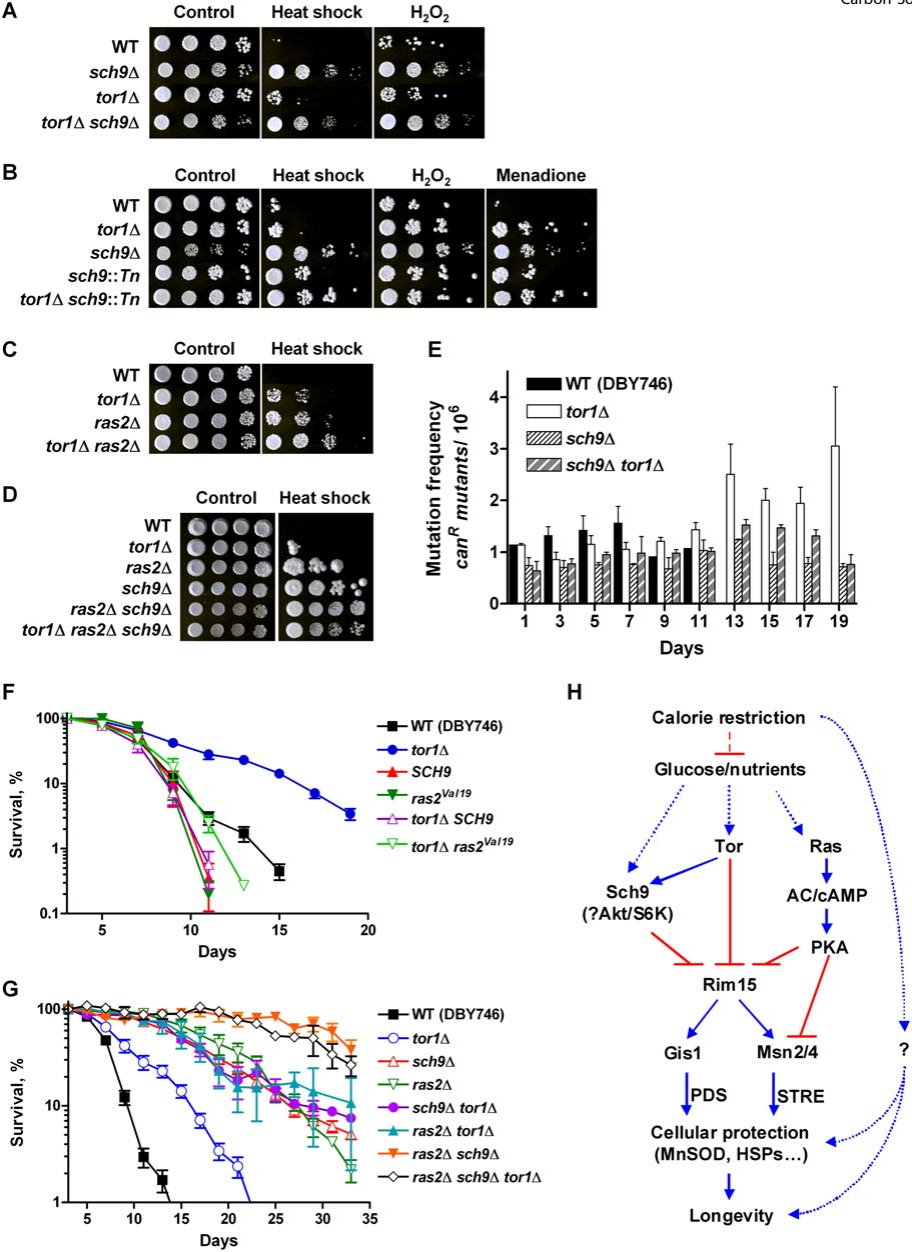
Genetic interactions between Sch9, Tor1, and Ras2 in regulating stress resistance and life span. (A–D) Day 3 wild type (DBY746) and cells lacking Tor1, Sch9, or Ras2 were challenged with heat shock (55°C: A, 105 min; B, 75 min; C, 150 min; and D, 120 min) or oxidative stresses (H_2_O_2_, 100 mM for 60 min; or menadione, 250 µM for 30 min). (E) Mutation frequency over time measured as Can^r^ mutants per million cells. The average of four experiments is shown. Error bars represent SEM. (F) Chronological survival in minimal complete medium (SDC) of wild type, *tor1*Δ, and mutants overexpressing either *SCH9* or constitutively active Ras2 (*ras2^Val19^*). (G) Chronological survival of wild type and mutants lacking Tor1, Sch9, Ras2 or combinations. The data represent average of at least 4 experiments. Error bars show SEM. For mean life span calculated from non-linear curve fitting see Table S1. (H) Longevity regulatory pathways in yeast. The nutrient sensing pathways controlled by Sch9, Tor, and Ras converge on the protein kinase Rim15. In turn, the stress response transcription factors Msn2, Msn4, and Gis1 transactivate stress response genes and enhance cellular protection, which lead to life span extension. Pro-longevity effects of CR are partially mediated by Sch9, Tor, and Ras, and may also require additional yet-to-be indentified mechanism(s).

Using a genetic approach, we examined the relationship between Sch9, Tor1, and Ras2 in regulating cellular protection against stress and life span. The effects on life span and stress resistance caused by deficiency in Tor1 activity are less robust than those observed in the strains lacking Sch9 or Ras2. We did not observe any significant difference in mean lifespan or stress resistance between *sch9*Δ and the *tor1*Δ *sch9*Δ double knockout strains (Figure 1A, G, and Table S1). By contrast, the deletion of *TOR1* in a mutant carrying a transposon insertion in the promoter region of *SCH9,* which only reduces *SCH9* expression [4], caused a further increase of resistance to heat and to the superoxide-generating agent menadione, but not to H_2_O_2_ (Figure 1B), suggesting that the lack of *TOR1* contributes to the further inactivation of the Sch9 pathway. This result is in agreement with the recent study showing that Sch9 is a direct target of rapamycin-sensitive Tor complex I (TORC1) [26]. In fact, reducing the TORC1 activity either by deleting *TCO89,* which encodes a TORC1 component, or by rapamycin treatment increased cell resistance to heat and H_2_O_2_ (Figure S1).

Since Sch9 activity is associated with an age-dependent increase of mutation frequency [23], we examined the interaction between Sch9 and Tor1 in the regulation of genomic instability during chronological aging. Whereas the *tor1*Δ mutant was slightly less susceptible than wild type cells to genomic instability (measured as age-dependent frequency of mutations of the *CAN1* gene) between day 1 and 7, there was no additive effect of *TOR1* and *SCH9* double deletion in the mutation frequency compared to that of the *sch9*Δ mutant **(** Figure 1E). Overexpression of *TOR1* only slightly reduced the stress resistance phenotype of *sch9*Δ (data not shown). However, resistance to stress and life span extension of *tor1*Δ was abolished by overexpressing *SCH9* (Figure 1F and data not shown). Taken together, these data are in agreement with a shared signaling pathway between Tor and Sch9 in life span regulation and suggest an upstream role of Tor1 in Sch9 signaling (Figure 1H).

Both Tor and Ras/cAMP-PKA pathways are known to regulate stress-responsive (STRE) genes [27]. Elevating Ras activity by ectopically expressing constitutively active Ras2 (*ras2^Val19^*) reversed the life span extension and the stress resistance of *tor1*Δ mutants (Figure 1F and data not shown). Conversely, deletion of *RAS2* had an additive effect to *tor1*Δ with respect to stress resistance but not life span (Figure 1C, G, and Table S1), suggesting an overlap in longevity modulation by Tor1 and Ras2.

We have previously shown that longevity regulation controlled by Tor1, Sch9 and Ras2 converges on the protein kinase Rim15 [24]. Rim15 positively regulates stress response transcription factors (TFs) Msn2/4 and Gis1, which activate genes involved in cellular protection. Interestingly, enhancement of stress resistance and life span extension associated with Ras2 deficiency requires both the STRE-binding TFs Msn2/4 and PDS-binding Gis1, whereas the *sch9*Δ-mediated longevity regulation mainly depends on the latter [4],[24]. These results indicate that the common downstream effectors are differentially modulated by the Sch9 and Ras2. In fact, the *ras2*Δ *sch9*Δ double knockout cells exhibited higher stress resistance than either of the single deletion mutants (Figure 1D). It also showed a 5-fold increase in mean life span compared to wild type cells (Figure 1G and Table S1). The triple *sch9*Δ *ras2*Δ *tor1*Δ deletion mutant, however, did not show any further increase of life span or stress resistance (Figure 1D, G, and Table S1). These results depict a life-span regulatory network composed of parallel but partially connected signaling pathways controlled by Tor/Sch9 and Ras (Figure 1H).

### Gene Expression Profiles of Long-Lived Mutants

**Figure 2:**
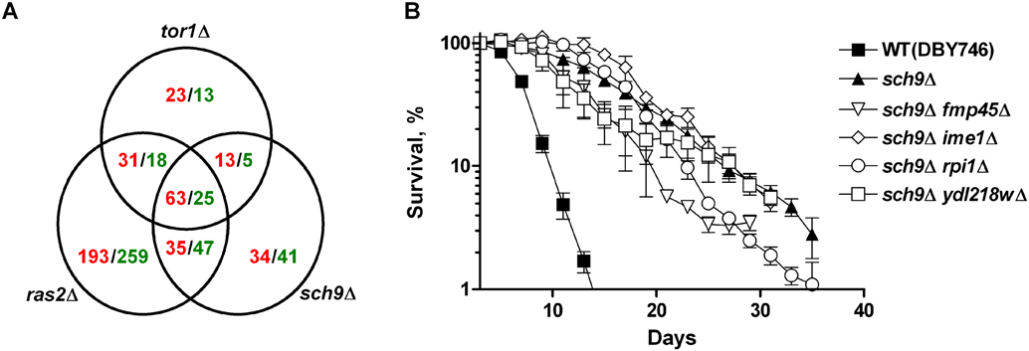
Gene-expression profiles of long-lived mutants. (A) Venn diagram of genes up- or down-regulated more than 2-fold in the *tor1*Δ, *sch9*Δ, and *ras2*Δ mutants, at day 2.5, compared to wild type cells (DBY746). Microarray analyses were carried out in triplicates. The number of up- or down-regulated genes was shown in red or green, respectively. For the significance of overlapping in up- and down-regulated genes see Table S3. (B) Life span of mutants with deletions of genes most upregulated in long-lived mutants in the *sch9*Δ background. Data represent mean and SEM of pair matched, pooled experiments. For mean life span calculated from non-linear curve fitting see Table S1.

To identify the mediators of life span extension downstream of the Tor/Sch9 and Ras pathways, we carried out DNA microarray analyses for all three long-lived mutants: *sch9*Δ, *tor1*Δ and *ras2*Δ. Total RNA was extracted from 2.5 day-old cultures of long-lived mutants and wild type cells. This age was selected to avoid both the noise that may arise from a small fraction of cells that are still dividing at younger ages (day1–2) and the general decrease in metabolism and consequently in gene expression that normally occurs at older ages (day 4–5) [22]. The cRNA obtained from total RNA was hybridized to gene chips that allow the detection of 5841 of the 5845 genes present in *S. cerevisiae*. Three independent populations of each genotype were analyzed. A total of 800 genes showed a greater than 2-fold change in expression relative to those in wild type cells. Among these, 63 genes were consistently up-regulated more than 2-fold in all three mutants, and 25 genes were consistently down-regulated **(** Figure 2A, for complete microarray data, see Table S2). The mRNA levels of seven of the most up-regulated and one most down-regulated genes in both the *tor1*Δ and *sch9*Δ mutants were confirmed by quantitative RT-PCR and/or Northern blot (Figure S2). Based on the pair-wise comparison of the long-lived mutants, the up- and down-regulation of genes in these long-lived mutants are significantly overlapping, suggesting that the Ras, Tor, and Sch9-centered longevity regulatory network controls a common set of down-stream genes (Table 1 and Table S3). To identify features common to the three long-lived mutants, we performed a gene ontology (GO) analysis of the microarray data by Wilcoxon rank test. Although the data point to common changes in all 3 long-lived mutants, the GO category analysis indicated a divergence in expression pattern between *ras2*Δ and the other two mutants (Table S4), which is in agreement with our genetic analysis of two parallel signaling pathways controlled by Sch9 and Ras2, and is consistent with the role of Sch9 and Tor1 in the same life span regulatory pathway (Table 1 and Figure 1H) [24].

**Table 1:** Gene ontology (GO) analysis of expression profiles of long-lived mutants.

***Positively affected GO categories***				***sch9***Δ		***tor1***Δ		***ras2***Δ	
**GO** *	**GO ID**	**Gene #**	**Annotation**	***p***	***q***	***p***	***q***	***p***	***q***
C	GO:0005842	93	cytosolic large ribosomal subunit	**0.00E+00**	**0.00E+00**	**0.00E+00**	**0.00E+00**	**1.64E-12**	**2.37E-10**
C	GO:0005843	63	cytosolic small ribosomal subunit	**0.00E+00**	**0.00E+00**	**0.00E+00**	**0.00E+00**	**7.49E-09**	**6.49E-07**
P	GO:0016125	37	sterol metabolism	**5.65E-03**	6.20E-02	**7.50E-03**	7.56E-02	**7.51E-05**	**2.32E-03**
P	GO:0046365	33	monosaccharide catabolism	**1.32E-03**	2.01E-02	**2.94E-05**	**1.02E-03**	**8.81E-06**	**3.81E-04**
***Negatively affected GO categories***				***sch9***Δ		***tor1***Δ		***ras2***Δ	
**GO** *	**GO ID**	**Gene #**	**Annotation**	***p***	***q***	***p***	***q***	***p***	***q***
C	GO:0005762	43	mitochondrial large ribosomal subunit	**1.56E-19**	**3.32E-17**	**1.13E-20**	**4.29E-18**	**1.34E-20**	**4.29E-18**
C	GO:0005763	34	mitochondrial small ribosomal subunit	**6.94E-13**	**4.93E-11**	**3.17E-13**	**2.54E-11**	**4.83E-14**	**4.41E-12**
C	GO:0016591	74	DNA-directed RNA polymerase II, holoenzyme	**1.61E-05**	**2.29E-04**	**9.05E-05**	**8.65E-04**	**4.97E-10**	**2.27E-08**
C	GO:0000502	46	proteasome complex	**3.92E-04**	**2.56E-03**	**4.51E-03**	1.72E-02	**1.35E-08**	**4.79E-07**
C	GO:0005743	158	mitochondrial inner membrane	**2.64E-16**	**2.82E-14**	**3.56E-17**	**5.70E-15**	**3.14E-09**	**1.34E-07**
F	GO:0008080	37	N-acetyltransferase activity	**6.89E-03**	2.32E-02	**6.43E-03**	2.25E-02	**3.16E-04**	**2.20E-03**
P	GO:0016570	59	histone modification	**1.56E-03**	**7.85E-03**	**2.16E-04**	**1.64E-03**	**7.30E-06**	**1.14E-04**
P	GO:0006365	67	35S primary transcript processing	**1.93E-03**	**9.16E-03**	**3.84E-06**	**7.23E-05**	**4.05E-03**	1.59E-02
P	GO:0007005	95	mitochondrion organization and biogenesis	**6.62E-05**	**7.02E-04**	**1.32E-04**	**1.07E-03**	**4.51E-06**	**8.02E-05**
P	GO:0016044	31	membrane organization and biogenesis	**2.09E-03**	**9.85E-03**	**1.38E-03**	**7.18E-03**	**9.74E-03**	3.06E-02
P	GO:0006626	47	protein-mitochondrial targeting	**8.33E-06**	**1.27E-04**	**1.46E-06**	**3.11E-05**	**4.04E-04**	**2.59E-03**
P	GO:0009060	82	aerobic respiration	**2.66E-08**	**8.96E-07**	**4.73E-09**	**1.78E-07**	**1.32E-06**	**3.01E-05**
P	GO:0006119	46	oxidative phosphorylation	**7.03E-07**	**2.04E-05**	**9.01E-07**	**2.31E-05**	**1.57E-04**	**1.24E-03**
P	GO:0006118	31	electron transport	**1.22E-04**	**1.03E-03**	**1.01E-04**	**9.28E-04**	**4.29E-03**	1.65E-02

Significantly up-or down-regulated categories were shown in bold (*p*<0.01). *q*-value was also calculated to correct the multi-testing error.

*: GO categories: C, Cellular component; F, molecular function; and P, biological process.

### Metabolic Changes Associated with Longevity-Extension

Gene expression profile comparison between long-lived mutants and wild type cells reveals a consistent down-regulation of the genes encoding mitochondrial proteins, including proteins functioning in the TCA cycle and oxidative phosphorylation, mitochondrial ribosomal proteins, as well as proteins targeted to mitochondria (Table S5). The expression of glycolytic/fermentative genes, but not of gluconeogenic genes, was instead up-regulated **(** Table S5C). Interestingly, several genes coding for high-affinity glucose transporters or putative glucose transporters, known to be inhibited by high glucose concentrations [28], were up-regulated indicating that the long-lived mutants may have entered a starvation-like mode in which glucose uptake is maximized **(** Table S5D). Considering that the extracellular glucose was exhausted in mutants as well as wild type cells by day 1–2 (data not shown), the major substrate available for fermentation by day 2.5 is probably glycogen, which is normally accumulated by yeast in the late phases of exponential growth [29].

Genes involved in stationary phase survival, sporulation, meiosis, and stress response (*FMP45*, *GRE1*, *IME1*, *RPI1*, *SPS100*, and *TAH1*) were among the most upregulated genes in all three long-lived mutants (Table S2). To test their contribution to life span extension and stress resistance in long-lived mutants, we originated a set of double mutants carrying the deletion of *SCH9, RAS2* or *TOR1* in combination with that of one of the most up-regulated genes. Whereas the deletion of either *FMP45* or *YDL218W* slightly reduced the mean life span of the *sch9*Δ mutants (Figure 2B), they have no effect on *ras2*Δ mutants (Figure S3B). The deletion of *IME1*or *RPI1* did not affect either the stress resistance or the life span extension caused by the lack of Sch9 (Figure 2B and data not shown). Deletion of *YLR012C*, the most down-regulated gene, did not affect significantly the life span or the stress resistance of the cell (Figure S3A).

Several genes coding for proteins that function in the ergosterol biosynthesis were up-regulated in the long-lived mutants (Table S7). Ergosterol is the predominant sterol in yeast and is structurally closely related to cholesterol. Besides being a structural component of the cellular membrane, ergosterol affects phospholipid synthesis, lipid rafts formation, signal transduction as well as aerobic energy metabolism [30]. The deletion of either *ERG4* or *ERG28* caused a small decrease in both heat and oxidative stress resistance in the *sch9*Δ mutants (Figure S3D). However, the deletion of *ERG5*, the most up-regulated ergosterol biosynthesis gene in our microarray analysis, did not reverse longevity extension or reduce stress resistance associated with the *sch9*Δ mutants (data now shown**)**. Notably, the ergosterol biosynthetic genes that were upregulated in all three long-lived mutants are those involved in converting squalene to ergosterol, which require molecular oxygen and often involve oxidation of NADPH to NADP^+^ (Table S7). The upregulation may reflect a hypoxic environment during the post-diauxic phase survival of these long-lived mutants and suggests a link between redox state of the cell and survival. Taken together, these results indicate that the single deletion of many genes among the most up-regulated in long-lived mutants has little effect on life span.

### Increased Expression of Glycerol Biosynthetic Genes in Long-Lived Mutants

**Figure 3:**
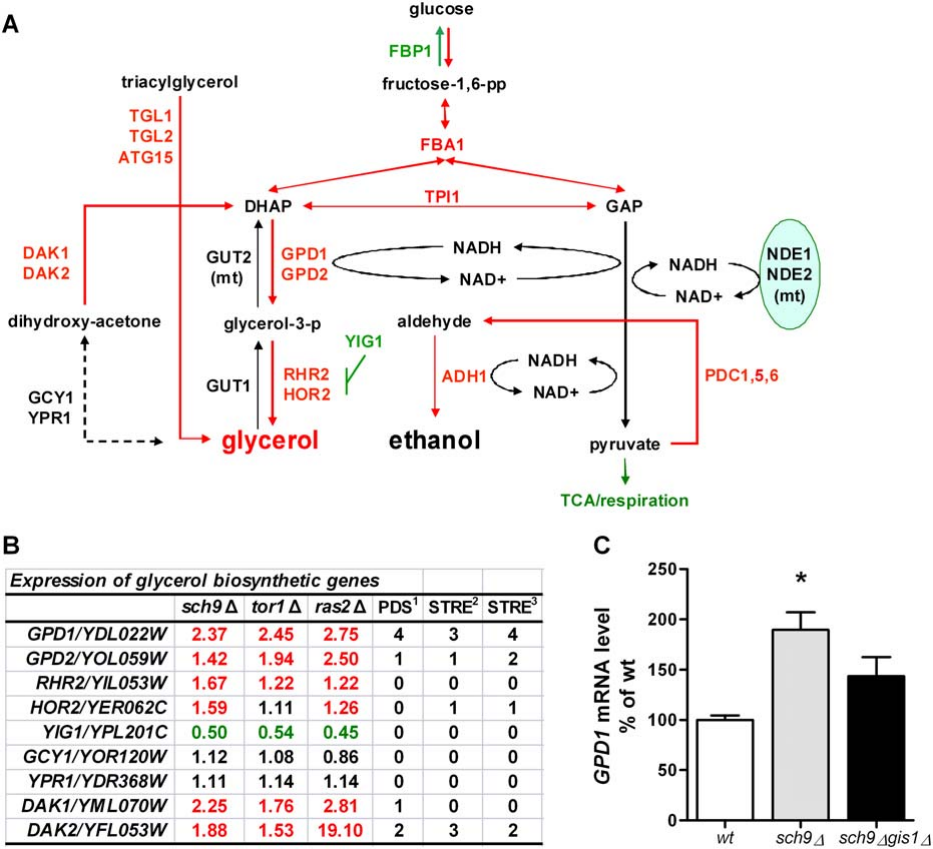
Expression of the glycerol biosynthesis genes. (A) Schematic representation of glycerol metabolism. For illustration purpose, genes up- or down-regulated more than 20%, compared to wild type (DBY746) in all three long-lived mutants, are labeled in red or green, respectively. (B) Fold change in expression levels of glycerol biosynthetic genes in *sch9*Δ, *tor1*Δ, and *ras2*Δ mutants compared to wild type at day 2.5 (for complete microarray data, see Table S2 and Table S6). Data were expressed in fold change, WT = 1. Gene expression levels that are 20% higher or lower than wild type cells are marked in red or green. ^1^ Predicted PDS motif: AWAGGGAT; ^2^ predicted STRE motif: ARGGGG; ^3^ predicted STRE motif: AGGGG. (C) Real time quantitative PCR analysis of *GPD1* mRNA level *sch9*Δ and *sch9*Δ *gis1*Δ cells at day 2.5. Data represent mean and SEM, n = 4. * *p*<0.05, *t-*test, two-tailed, *sch9*Δ *vs.* WT.

In addition to the lower expression of TCA cycle and respiratory genes and higher expression of glycolytic/fermentative genes, we also observed an up-regulation of the genes implicated in the metabolism of glycerol, a byproduct of the overflow metabolism when there is enhanced glycolytic flux and limited respiration capacity (Figure 3A and B). Significant up-regulation of genes involved in glycerol metabolism (21 genes, Table S6) was observed in *sch9*Δ and *ras2*Δ mutants (*p*-value of 0.0058 and 0.0142, Wilcoxon rank test, one-sided, respectively). In yeast, glycerol is produced from either triacylglycerol or dihydroxy-acetone-phosphate (DHAP), a glycolysis intermediate (Figure 3A). Whereas the genes encoding the lipases responsible for the hydrolysis of triacylglycerol were slightly up-regulated, *GPD1* and *GPD2*, encoding the key enzymes required for glycerol production from DHAP, showed higher levels of expression in all the long-lived mutants (Figure 3B and C), suggesting that part of the glucose utilized by these mutants is redirected towards glycerol biosynthesis. A search in the 800 bp upstream promoter region of the glycerol biosynthesis genes revealed that *GPD1*, *GPD2*, *HOR2*, *DAK1*, and *DAK2* contain the DNA binding elements of Gis1- and Msn2/4 (Figure 3B), stress resistance transcription factors downstream of Tor1, Sch9, and Ras2 [24]. Furthermore, the up-regulation of glycerol biosynthesis genes were partially reversed in *sch9*Δ *gis1*Δ double mutants (Figure 3C; Figure S2J and K), indicating that regulation of glycerol biosynthesis is part of the regulatory repertoire of Sch9 signaling.

**Figure 4:**
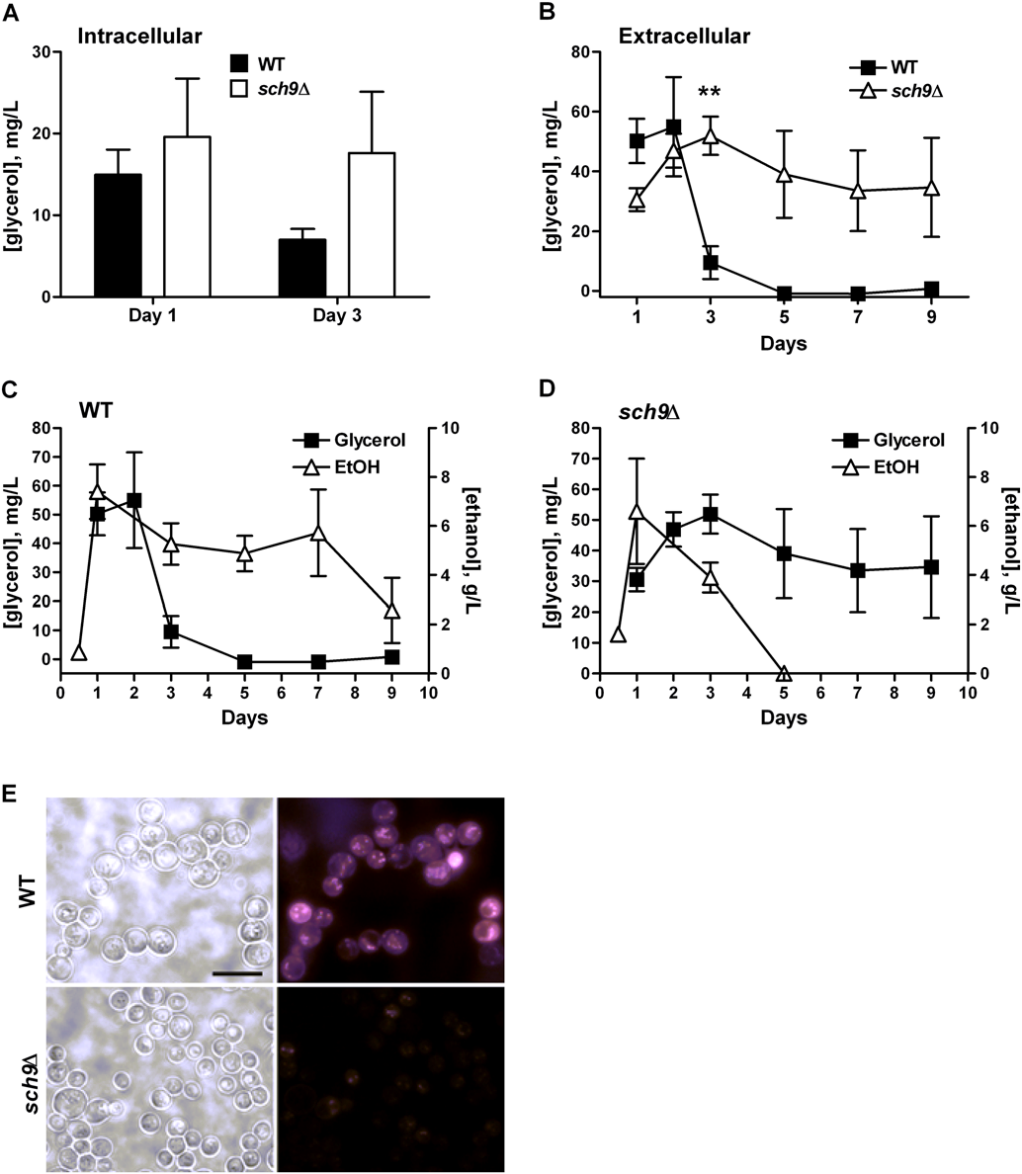
Sch9 deficient mutants metabolize ethanol and accumulate glycerol. (A) Intracellular glycerol contents of wild type (DBY746) and cells lacking Sch9 were measured on day 1 and day 3. Data represent mean and SEM of 8 cultures analyzed. (B) Glycerol concentration in the medium of wild type and *sch9*Δ cultures. Data represent mean and SEM of 5–7 cultures analyzed. ** *p*<0.01, unpaired *t*-test, two-tailed, *sch9*Δ *vs.* WT. (C–D) Glycerol and ethanol concentrations in the medium of wild type (C) and *sch9*Δ (D) cultures. Data represent mean and SEM of 3–5 cultures analyzed. (E) Nile red staining of neutral lipids of day 1 wild type and *sch9*Δ mutants. Nile red staining is shown on the right, and phase contrast left. Bar, 10 µm.

In fact, higher level of intracellular glycerol was observed in the *sch9*Δ mutants compared to that in wild type cells at day 3 (Figure 4A). In wild type cells the level of extracellular glycerol reached a peak at day 2 but was mostly depleted by day 3. In the *sch9*Δ culture, however, a much elevated level of glycerol was measured in the medium up to day 9 **(** Figure 4B). By contrast, ethanol produced during the exponential growth, and most likely in the post-diauxic phase as well, was depleted early in *sch9*Δ mutants but not in wild type cells (Figure 4C and D) [23], suggesting a metabolic switch from biosynthesis and release of ethanol in wild type cells to that of glycerol in *sch9*Δ mutants. Glycerol accumulation could be accompanied by the depletion of other carbon sources as well. Nile red staining of the lipid body indicated that the levels of triacylglycerol and other neutral lipids in *sch9*Δ mutants were consistently lower compared to that in wild type cells across all ages (Figure 4E and data not shown), which is in agreement with a modest but consistent increase of lipolytic enzyme mRNA levels (Table S6). Accumulation of extracellular glycerol also occurred in *tor1*Δ and *ras2*Δ mutants, but was lower than that observed in *sch9*Δ mutants (Figure S4).

### Glycerol Biosynthesis Genes Are Required for Life Span Extension in *sch9*Δ

**Figure 5:**
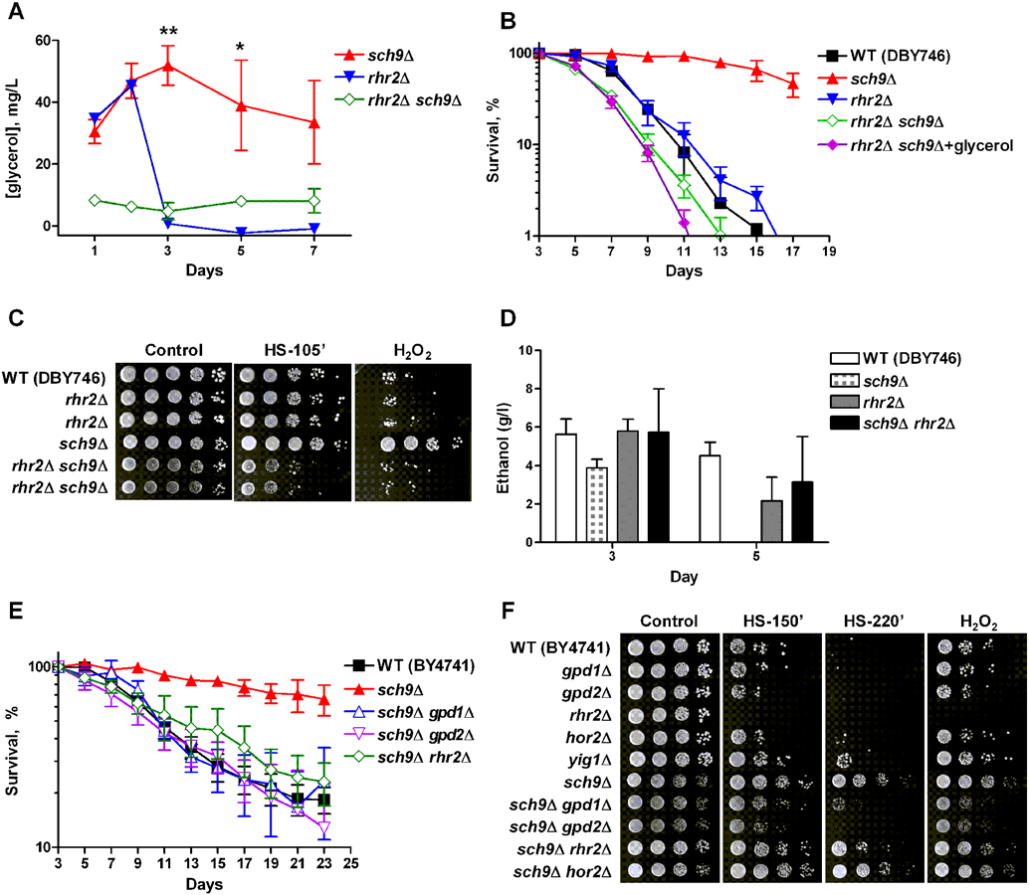
Deletion of glycerol biosynthesis genes reverses life span extension and stress resistance associated with Sch9 deficiency. (A) Glycerol concentration in the medium. Data represent mean and SEM of 4 cultures analyzed. * *p*<0.05, ** *p*<0.01, unpaired *t*-test, two tailed, *sch9*Δ *vs. rhr2*Δ *sch9*Δ. (B) Life span of wild type (DBY746), *sch9*Δ, *rhr2*Δ, and Sch9-deficient mutants lacking Rhr2. Glycerol (1%, final concentration) was added to the one day-old *rhr2*Δ *sch9*Δ culture. Data represent mean and SEM of 4–5 cultures analyzed. (C) Day3 cells were exposed to heat shock (55°C for 105 min) or H_2_O_2_ (150 mM for 60 min). Strains shown are wild type (DBY746), *rhr2*Δ, *sch9*Δ, and *rhr2*Δ *sch9*Δ. (D) Ethanol concentration in the medium. Data represent mean and SEM, n = 3. Ethanol in *sch9*Δ culture at day 5 was at the lower detection limit of the assay. (E) Life span of wild type (BY4741), *sch9*Δ, and Sch9-deficient mutants lacking Gpd1, Gpd2, or Rhr2. Data represent mean and SEM of 3 experiments. (F) Heat shock (55°C) and oxidative stress (H_2_O_2_, 500 mM, 60 min) resistance of day 3 mutants lacking glycerol biosynthesis genes (in the BY4741 genetic background).

To further examine the role of glycerol biosynthesis in life span regulation, we generated strains lacking Rhr2, the yeast _DL_-glycerol-3-phosphatase, in the *sch9*Δ background. The *rhr2*Δ *sch9*Δ double mutant failed to accumulate glycerol extracellularly (Figure 5A). Deletion of *RHR2* abolished the life span extension as well as the resistance to heat and oxidative stresses associated with the *sch9*Δ mutants in the DBY746 genetic background (Figure 5B and C). Interestingly, ethanol was still present in the medium of *rhr2*Δ *sch9*Δ mutants at day 5 (Figure 5D), when ethanol is mostly depleted in the *sch9*Δ culture (Figure 4D), although in 3 independent cultures (3 independent isolates) assayed, we observed high variation in ethanol concentration. Utilizing the yeast KO collection (BY4741 genetic background), we deleted *SCH9* in strains lacking key glycerol biosynthetic genes. Deficiency in either of the NAD-dependent glycerol 3-phosphate dehydrogenase genes, *GPD1* or *GPD2*, did not cause a significant life span change in wild type BY4741 cells (Figure S5A). However, the deletion of either *GPD1* or *GPD2*, led to the reversion of the longevity extension associated with Sch9 deficiency (Figure 5E). Similarly, the deletion of *RHR2* abolished the life span extension in the *sch9*Δ mutant (Figure 5E). In contrast, lack of Hor2, a redundant isoenzyme of _DL_-glycerol-3-phosphatase, did not affect the life span of the *sch9*Δ mutant (data not shown). The difference between these two isoenzymes may be explained by the fact that Rhr2 is the predominant isoenzyme in the cell [31]. In agreement with the major role of Rhr2, the mRNA level of *YIG1*, coding for an inhibitor of Rhr2 [32], was down-regulated in all long-lived mutants (Figure 3B). Notably, the life span of *rhr2*Δ mutants in the BY4741 genetic background was similar to that of wild type cells, although some *rhr2*Δ cultures showed regrowth/gasping (data not shown) [33].

Cells lacking both Rhr2 and Hor2 have been shown to be hypersensitive to the superoxide anion generator, paraquat, suggesting a role for glycerol biosynthesis in cellular protection beyond osmotic stress [34]. We also tested the role of glycerol biosynthetic genes in the stress resistance of sc*h9*Δ mutants in the BY4741 background. Hypersensitivity to heat and hydrogen peroxide-induced oxidative stress was observed in the *RHR2*-null strain, but not in *gpd1*Δ, *gpd2*Δ, or *hor2*Δ mutants in the BY4741 background (Figure 5F). Furthermore, cells lacking Yig1, the Rhr2 inhibitor, were slightly more resistant to stress compared to wild type cells (Figure 5F). The stress resistance phenotype of *sch9*Δ mutants was reversed by the deletion of *GPD1* or *GPD2*, but not of *RHR2* or *HOR2* (Figure 5F). There appears to be redundancy in glycerol-mediated response to stress such that deficiency of one enzyme can be compensated by activation of others in the glycerol biosynthesis pathway. Deletion of *SCH9* greatly enhanced stress resistance to heat and H_2_O_2_ of *rhr2*Δ mutants (Figure 5F), possibly due to the upregulation of the Hor2 level. Since glycerol phosphatases (Rhr2 and Hor2) are not the rate-limiting enzymes for glycerol production [34], the upregulation of Gpd1 and Gpd2 may also contribute to the rescue of the *rhr2*Δ stress sensitive phenotype in cells lacking *SCH9*. A similar redundancy exists between Gpd1 and Gpd2. Although little or no effect was seen in either of the single deletion mutants, *gpd1*/*2*Δ double knockout strain is hypersensitive to heat and hydrogen peroxide treatment (data not shown). The triple *sch9*Δ *gpd1*Δ *gpd2*Δ mutant showed severe growth defects and low saturation density in the liquid culture, which prevented us from utilizing this mutant for epistatic studies (data not shown). Taken together, these results underscore the importance of glycerol biosynthesis in promoting cellular protection and life span extension in the *SCH9* deficient mutants.

**Figure 6:**
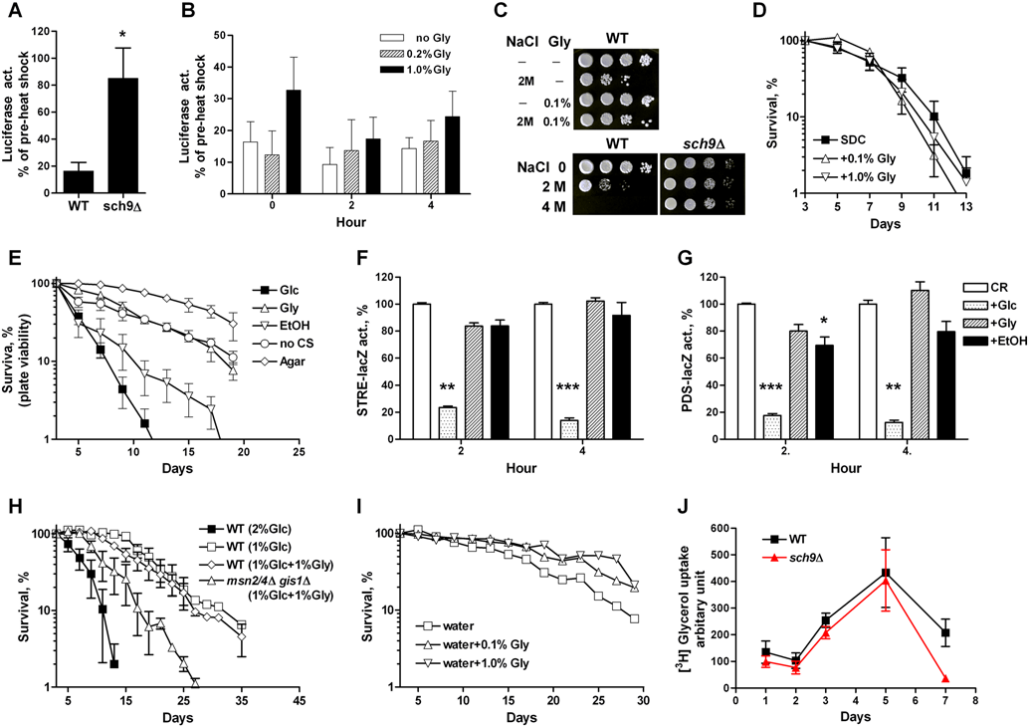
Role of glycerol in stress resistance and life span. (A) Day 3 wild type (DBY746) and *sch9*Δ mutants expressing bacterial heat-sensitive luciferase were subject to heat stress (42°C for 60 min). Data represent mean and SEM, n = 3. * *p*<0.05, unpaired *t-*test, two tailed. (B) Recovery of luciferase activity after heat stress (42°C for 60 min) in wild type cells pre-treated with glycerol (with concentrations indicated) for 30 min. Data represent mean and SEM, n = 3. (C) Day 3 wild type and *sch9*Δ mutants grown in SDC were washed 3 times with water and exposed to high concentrations of NaCl with or without glycerol for 24 hours. The cells were then washed 3 times to remove the salt, serially diluted, and spotted on to YPD plate. (D) Chronological survival of wild type cells grown in SDC supplemented with glycerol. Data represent mean and SEM, n = 3. (E) Chronological survival in the presence of various carbon sources using the *in situ* viability assay. Day 1 SDC wild type cultures were diluted and plated onto SC-Trp plates (no carbon source, CS), SC-Trp plates supplemented with glucose (2%, Glc), ethanol (0.8%, EtOH), or glycerol (3%, Gly), or agar plates (extreme CR). Data represent mean and SEM, n = 3–8. (F, G) One-day old SDC wild type cells were switched to water and incubated for 4 hours. The STRE- (F) and PDS-lacZ (G) activities were measured 2 and 4 after the addition of glucose, glycerol, or ethanol (final, 0.8%) and shown as the percentage of CR. Data shown are mean and SEM of four independent samples assayed. *, *p*<0.05; **, *p*<0.01; ***, *p*<0.001, Tukey's multiple comparison test, carbon source added *vs.* CR. (H) Chronological survival of wild type and *msn2*Δ *msn4*Δ *gis1*Δ mutants grown in normal (SC+2% glucose), reduced glucose (SC+1% glucose), or glucose/glycerol (SC+1%+1%) medium. Data represent mean and SEM of 4 cultures analyzed. (I) Day 3 wild type cells grown in SDC medium were washed three times with water and incubated in water (extreme CR/starvation) with or without glycerol (0.1% or 1%). Plot shows a representative experiment (mean of duplicates) repeated three times with similar results. (J) Yeast grown in SDC was sampled (1 ml) at indicated time points. [1,2,3-^3^H] Glycerol (ARC, Inc) was added to the aliquot and incubated at 30°C with shaking for 24 hours. Cells were then washed three times with water. The cellular [^3^H]-content was determined by scintillation counting (Wallac 1410, Pharmacia) and normalized to cell number (viability by CFU). Data represent mean and SEM of 4 cultures analyzed.

Glycerol can protect against stress in part because of its function as a chemical chaperone [35]–[37]. To test the role of glycerol in protecting against heat-induced protein misfolding, we examined the activity loss and recovery of a heat sensitive bacterial luciferase [38] in wild type and *sch9*Δ cells. Whereas exposing wild type cells to heat stress (42°C for 1 hour) led to a ∼80% reduction of luciferase activity, only a 20–40% loss of activity was observed in *sch9*Δ mutants (Figure 6A), which is consistent with the enhanced stress resistance phenotype of *sch9*Δ (Figure 1). However, pre-treatment of wild type cells with low concentration of glycerol had no protective effect on the heat-induced loss and the recovery of luciferase activity (Figure 6B), indicating the heat resistance phenotype of *sch9*Δ does not depend on the short-term exposure to extracellular glycerol. Similar results were obtained in the BY4741 genetic background (Figure S5B and C).

Intracellular accumulation of glycerol also contributes to protection against osmotic stress [37],[39]. Addition of 0.1% of glycerol to the medium slightly enhanced the resistance to osmotic stress of wild type yeast (Figure 6C). When exposed to high concentration of NaCl, the *sch9*Δ and *ras2*Δ mutants exhibited enhanced resistance to hyperosmolarity compared to the *tor1*Δ mutant, which in turn was better protected than wild type cells (Figure 6C and Figure S4B), suggesting that increased resistance against hyperosmolarity may be part of the general stress response shared by all long-lived mutants. These data are also consistent with the reports that high osmolarity growth conditions extend both RLS and CLS in yeast [40],[41]. With regard to life span, however, extracellular supplementation of glycerol (0.1% and 1%) to the wild type yeast culture at day 3, when the glycerol level is high in the long-lived *sch9*Δ mutants (Figure 4B), did not show any beneficial effect (Figure 6D).

### Glycerol Provides a Carbon Source without Blocking the Anti-Aging Effect of Calorie Restriction

Ethanol, as a carbon source, elicits pro-aging signaling and promotes cell death. Removing ethanol either by evaporation or by switching yeast cells from expired medium to water, which represents a condition of extreme calorie restriction/starvation, extends yeast chronological life span [23]. The metabolic switch to ethanol utilization and glycerol biosynthesis removes the detrimental effect of pro-aging carbon sources (glucose and ethanol) and creates an environment that mimics calorie restriction in the *sch9*Δ mutant culture (Figure 4D). To elucidate the role of different carbon sources on life span, we used an *in situ* assay to monitor chronological survival of yeast on plate [42], which allowed us to: a) study the effect of different carbon sources in the presence of all the other nutrients, b) control the exact amount of carbon source to which the cells are exposed over the whole experiment, similarly to the experimental conditions used for the RLS studies of calorie restriction.

The survival curve of approximately 200 wild type DBY746 cells plated onto SC-Trp plates supplemented with 2% glucose is reminiscent of that in the standard liquid medium paradigm (Figure 6E). Extreme CR/starvation (agar plate) or removal of carbon source from the SC-Trp plates leads to life span extension, which was partially reversed by the presence of low concentration of ethanol (Figure 6E) in agreement of our earlier findings [23]. The adverse effect of glucose on life span was also observed on the long-lived *sch9*Δ and *ras2*Δ cells (Figure S6C and D). Substitution of glucose with high level of glycerol (3%), however, did not trigger the pro-aging signaling as seen with glucose or ethanol (Figure 6E). Thus, the metabolic switch to glycerol biosynthesis in the long-lived *sch9*Δ mutants may represent a genetically induced “carbon source substitution” that can be as effective as calorie restriction in the regulation of protection.

Calorie restriction-induced cellular protection and life span extension in yeast depends on the protein kinase Rim15 and the activation of its downstream stress response transcription factors, which are negatively regulated by Sch9, Tor, and Ras [24]. Extreme CR/starvation, achieved by switching yeast to water, activates Msn2/4 and Gis1 transactivation, via the STRE and PDS elements, respectively [24]. Addition of glucose and, to a lesser extent, ethanol significantly represses CR-induced STRE- and PDS-driven LacZ reporter gene expression (Figure 6F and G). However, no reduction in STRE and PDS transactivation were observed when CR yeast were exposed to glycerol (Figure 6F and G). Similar to extreme CR/starvation, reduction concentration of glucose in the culture medium also extends yeast life span [43]–[47] and requires the Msn2/4 and Gis1 [24]. When yeast were grown in medium containing either low glucose (1%) or glucose/glycerol (1% each), a 1.5-fold increase in mean life span was observed compared to that in the standard medium (Figure 6H). This pro-longevity effect of the low glucose/glycerol diet was mostly dependent, as is that of calorie restriction, on the stress response transcription factors (Figure 6H). Notably, the beneficial effect of calorie restriction on longevity does not require glycerol biosynthesis. Cells deficient of *RHR2* still lived long when cultured in reduced glucose or incubated in water (Figure S6A and B).

The metabolic switch in the *sch9*Δ mutants may not only remove the pro-aging/death signaling from glucose/ethanol or other carbon sources but also produce a carbon source, glycerol, for long-term survival. We switched wild type cells from the ethanol-containing medium to water containing 0.1% glycerol. A small extension of life span was observed in addition to that of extreme calorie restriction (Figure 6I), suggesting that glycerol may provide nutritional support or additional protection under the starvation condition. In fact, we show that yeast cells actively uptake the exogenous [1,2,3-^3^H] glycerol during the post-diauxic phase, entered by *S. cerevisiae* after most of the extracellular glucose is depleted (Figure 6J and Figure S4C). The utilization of glycerol is also supported by our microarray analysis, which showed that the genes involved in the catabolic metabolism of glycerol are up-regulated under the extreme CR/starvation (water) condition in wild type cells (Table S6).

## Discussion

**Figure 7:**
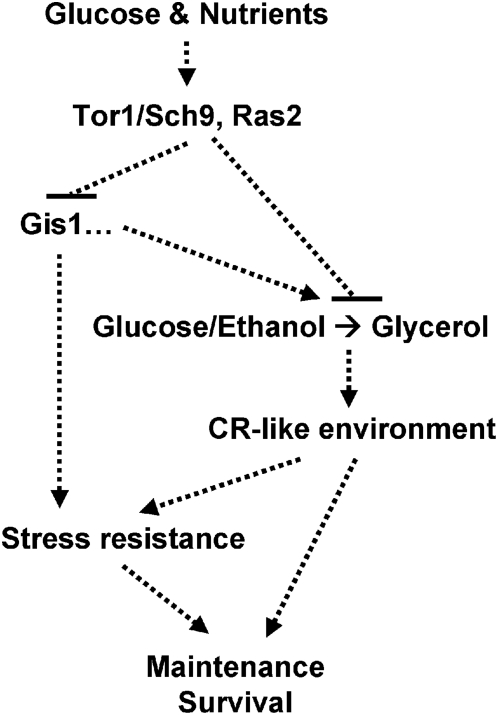
Inhibition of the Tor1/Sch9 and Ras pathways activates stress response transcription factors and glycerol biosynthesis. The metabolic changes in long lived mutants generate a calorie restriction-like environment, which contributes to enhanced stress resistance and extended life span.

Model organisms such as yeast, worms, and flies have been instrumental in the discovery of life span regulatory pathways that have a common evolutionary origin. Among these, the insulin/IGF-I-like pathways control longevity in organisms as phylogenetically distant as yeast and mice. Akt, Tor, and Ras function in the mammalian IGF-I signaling pathway and have been implicated in life span regulation in different model organisms [1],[48]. In this study, we show that longevity regulatory pathways control the shift from respiration to glycolysis and glycerol biosynthesis. This metabolic switch, which leads to the removal of pro-aging carbon sources and glycerol accumulation, creates an environment in the *sch9*Δ culture that mimics calorie restriction (Figure 7).

The genetic and genomic data revealed two parallel longevity signaling pathways controlled by Tor1/Sch9 and Ras, in agreement with our previous work [4]. The beneficial effects of reduced activities of both pathways is additive (Figure 1D and G), and the *sch9*Δ *ras2*Δ double mutant is one of the longest lived genetic mutants [49]. In agreement with the genetic data, the gene expression profile of the day 2.5-old *ras2*Δ mutant shows that approximately 67% of the genes differentially expressed are not significantly changed in the other two mutants (Figure 2A). Our genetic analysis of the interactions between the Tor, Sch9 and Ras2 indicates a stronger overlap between the Tor1 and Sch9 pathways in the regulation of stress resistance, longevity, and age-dependent genomic instability. It also suggests that TORC1 functions upstream of Sch9 in the regulation of these readouts in agreement with what has been proposed by others [50] and with the demonstration of the direct phosphorylation of Sch9 by TORC1 [26]. Our microarray analysis indicates similarities but also differences between the set of genes controlled by Tor and Ras. On the one hand, *TOR1* deletion further increased the heat-shock resistance of *ras2*Δ mutants, and on the other hand no additional life span extension was observed. Furthermore, the overexpression of constitutively active Ras2 abolished CLS extension associated with deficiency of *TOR1*, suggesting an overlapping of the two pathways and possibly an upstream role of TORC1.

Despite the higher degree of differential expression profile observed in *ras2*Δ mutants, there are remarkable similarities in the expression pattern of genes involved in key metabolic pathways in all three long-lived mutants. The genome-wide association (transcription factor binding motif enrichment test) and the genetic analyses indicate that longevity modulation by the Tor/Sch9 and Ras signaling depends on the protein kinase Rim15 and its downstream stress response transcription factors, Msn2/4 and Gis1 [24],[51]. The most striking result is that genes involved in glycolysis/fermentation are consistently upregulated, while mitochondrial related genes are down-regulated, in all three long-lived mutants, suggesting a cellular state that favors glycolysis and diminished mitochondrial functions including TCA cycle and oxidative phosphorylation. Part of our results may appear to contradict recent results showing that respiration is upregulated in the *tor1*Δ mutant [18]. This discrepancy may be explained by the difference in the time point of observation. Bonawitz and colleagues measured higher respiration rates in exponentially growing or day 1 *tor1*Δ cultures relative to wild type yeast. By day 2 this difference was no longer observed [18]. The role of respiration in replicative life span regulation is still unclear. On the one hand, increased respiration has been shown to mediate the beneficial effect of CR (0.5% glucose) [52]; on the other hand, growth on lower glucose-containing medium (0.05% glucose) can extend the replicative life span of respiratory-deficient yeast [15]. Moreover, a study from the Jazwinski's group indicated that respiration does not directly affect replicative longevity [53]. The different effect of respiration on life span may also be contributed to the experimental systems used for life span studies. The replicative life span analysis is mostly carried out on the solid rich YPD medium, where cells are constantly exposed to glucose and other nutrients. The energy required for growth is mainly derived from fermentation. In contrast, our chronological longevity studies are performed by monitoring population survival in a non-dividing phase in which fermentation is minimized [22].

The gene expression profiles of long-lived mutants showed the induction of key genes required for glycerol biosynthesis. High levels of extracellular and intracellular glycerol were detected in the *sch9*Δ culture and triglyceride catabolism appeared to contribute to glycerol generation (Figure 4). This shift towards the production of glycerol represents a fundamental metabolic change in the physiology of the long-lived mutants.

Genetic analysis performed by deleting genes required for glycerol biosynthesis in the *sch9*Δ mutant indicates that glycerol production is required for life span regulation (Figure 5). Increased glycerol biosynthesis may contribute to life span regulation through several distinct mechanisms. First, cells lacking Sch9 utilize glucose and ethanol and accumulate glycerol, a carbon source that does not promote aging and cell death. This metabolic change creates an environment that mimics calorie restriction. CR, achieved by either lowering glucose in growth medium or by removing ethanol, extends the yeast CLS [23],[24],[47]. Conversely, addition of low concentration of ethanol reverses life span extension induced by CR [23]. Here we show that cells lacking Sch9 deplete pro-aging carbon sources and activate glycerol biosynthesis. Whereas glucose and, to a lesser extent, ethanol promotes aging, glycerol acts as a “phantom carbon source” and does not inhibit the transactivation of stress response transcription factors Msn2/4 and Gis1, which play important roles in stress resistance and longevity modulation in both long-lived mutants and cells under calorie restriction (Figure 6) [24]. Since glycerol was taken up by the cells and caused a minor enhancement of survival under starvation conditions, it is likely that its uptake provides nutritional support for long term survival (Figure 6I). Second, production and accumulation of glycerol may contribute to cellular protection since glycerol enhances resistance to osmotic stress and functions as molecular chaperone stabilizing/renaturing the newly synthesized or heat-inactivated proteins. However, our present and past results indicate that Sch9 also down-regulates stress resistance systems independently of the generation of glycerol. For example, in the BY4741 background Sch9 deficiency increased the resistance to multiple stresses in mutants with defects in glycerol biosynthesis (Fig. 5F). Third, glycerol production may affect aging through the modulation of the redox balance of the cell, since its production contributes to the maintenance of NAD:NADH ratio [54]–[56]. Easlon *et al*. have recently shown that overexpression of the malate-aspartate NADH shuttle components extends yeast replicative life span [57]. The latter two mechanisms, however, are less likely to contribute significantly to chronological life span extension, as addition of exogenous glycerol to the culture had little or no effect on heat-induced protein inactivation (Figure 6B) or chronological survival in wild type cells (Figure 6D). Additionally, we overexpressed in wild type cells the bacterial NADH oxidase (NOX) or alternative oxidase (AOX), both of which increase NADH oxidation in yeast [58], did not significant alter the life span of the wild type cells (unpublished data).

In summary, we presented data showing enhanced expression of glycerol biosynthetic genes in three long-lived yeast mutants lacking *SCH9*, *TOR1*, or *RAS2*, whose homologs also play important roles in life span modulation in organisms ranging from worms, flies, to mammals. Our data also suggest that the switch to glycerol biosynthesis is required for life span extension in the *sch9*Δ mutants. We argue that the genetically induced carbon source substitution in the long-lived *tor1*Δ and *sch9*Δ cells creates a beneficial environment that mimics calorie restriction which, together with the intracellular regulation of stress resistance via transcription factors Gis1 and Msn2/4, results in life span extension and stress resistance (Figure 7). In light of the conservation of the longevity regulatory pathways and the role of calorie restriction in extending life span of a wide range of species, it will be important to investigate further the possibility of an anti-aging role for glycerol in higher eukaryotes.

## Materials and Methods

### *S. cerevisiae* Strains and Growth Conditions

Mutant strains used were originated in DBY746 (*MATα, leu2-3, 112, his3*Δ, *trp1-289, ura3-52, GAL^+^*) or BY4741 (*MATa, his3*Δ*1, leu2*Δ*0, met15*Δ*0, ura3*Δ*0*) by one-step gene replacement as described previously [59]. Strains overexpressing *SCH9* or *ras2^val19^* were generated by transforming DBY746 with plasmids pHA-*SCH9* (a gift from Dr. Morano University of Texas Medical School) or pMW101 (plasmid RS416 carrying *Cla* I-*ras2^val19^*-*Hind* III fragment of pMF100, a gift from Dr. Broach, Princeton University), respectively. Strains expressing a heat sensitive bacterial luciferase (Parsell, 1994) were generated by transforming yeast with plasmid pGPD-luxAB (Addgene.com). Yeast chronological life span was measured as described previously [22]. For *in situ* viability assay [42], day 1 SDC cultures of tryptophan auxotrophic strains were diluted and plated on to SC-Trp plates (∼200 cells/plate) with no carbon source, or supplemented with glucose (2%, as in standard SDC), glycerol (3%), or ethanol (0.8%, a concentration reached in wild type cultures during diauxic shift, see Figure 4C), or agar plates (starvation/water). Plates were incubated at 30°C for the duration of the experiment. Every two days, 0.5 ml of 2 mg/ml tryptophan was added to the plates. For plates without glucose, 1 ml of 5% glucose was added to the plates in additional to tryptophan. For agar plates, 1 ml of 2× YPD was added. Colony formation was monitored after 2–3 days incubation at 30°C.

### DNA Microarray Analysis and Real Time PCR

Total RNA were extracted from 2.5-day old wild type and mutants cultures (in SDC, n = 3) by the acid phenol method. The cRNA was hybridized to Affymetrix GeneChip Yeast 2.0 array to obtain the measurement of gene expression. Procedures for microarray data analysis have been described previously [51],[60]. The Gene Ontology (GO, ftp://genome-ftp.stanford.edu/pub/go/ontology/) data were organized as a directed acyclic graph, in which each node corresponded to a set of genes with specific annotations. Only the GO categories that were well annotated and contain ≥30 genes were included, which were defined as terminal informative GO (TIGO) categories: 44 cellular components, 53 molecular functions, and 109 biological processes. Wilcoxon rank test was performed to examine whether a TIGO category was significantly up- or down-regulated. Finally, q-values for each test were calculated to correct the multiple testing errors using the “qvalue” package [61]. For quantitative RT-PCR analysis, total mRNA was extracted from cells harvested from 2.5-day-old cultures. RNA was reverse-transcribed using RetroScript III (Invitrogen). Quantitative real time PCR was performed using the DNA Engine Opticon 2 (BioRad). Primers used are listed in Figure S2 legend. Gene expression levels were normalized to actin (*ACT1*) and expressed as the percentage of wild type.

### Stress Resistance Assays

Day 3 cells grown in SDC were used for stress resistance assay. For heat shock resistance, serial dilutions of cells were spotted onto YPD plates and incubating at 55°C (heat-shocked) for 60–150 min. Plates were then transferred to 30°C and incubated for 2–3 days. For oxidative stress resistance assays, cells were diluted to an OD_600_ of 1 in K-phosphate buffer (pH6), and treated with 100–200 mM of hydrogen peroxide for 60 minutes. Alternatively, cells were treated with 250 µM of menadione for 30 min in K-phosphate buffer (pH7.4). Serial dilutions of control or treated cells were spotted onto YPD plates and incubated at 30°C for 2–3 days. For osmotic stress resistance assay, cells were washed twice with water and resuspended in salt buffer (2 or 4 M NaCl). After incubating at 30°C for 24 h with shacking, cells were washed with water to eliminate salt, serially diluted, and then plated on to YPD plates. Plates were incubated 2–3 days at 30°C.

### Nile Red Staining

Cells (1 ml SDC culture) were washed once with PBS and resuspended in 1 ml PBS. 10 µl of Nile Red (0.1 mg/ml in acetone) was added to the cell suspension and incubated at room temperature, in the dark, for 5 min. Cells were washed once with PBS and imaged with a Leica fluorescent microscope.

### Glycerol and Ethanol Measurements

For intracellular glycerol content, cells were washed three times with water. Cell pellets from 1 ml culture were resuspended in 0.5 ml of Tris buffer (0.1 M, pH7.4) and, then, boiled for 5 min followed by a 30 sec spin to remove cell debris. The supernatant from the cell extract or the medium cleared of cells was used to determine intracellular or extracellular glycerol level, respectively. Glycerol concentration was measured using an UV-based glycerol assay kit (Boehringer Mannheim/R-Biopharm). The manufacturer recommended protocol was modified to adapt the assay to a 96-well plate format. Each sample was assayed in duplicates and data were fitted to standard curve generated by serial dilutions of stock glycerol. For intracellular glycerol measurement, glycerol concentrations were normalized to cell number based on viability assay. Ethanol concentration in medium was measured using the UV-based ethanol assay kit (Boehringer Mannheim/R-Biopharm) according to the manufacturer recommended protocol.

### Luciferase Assay

Heat inactivation of luciferase was measured as previously described (Parsell, 1994). Briefly, cells expressing heat-sensitive bacterial luciferase were subject to heat shock (42°C for 60 min). Ten minutes before the end of heat shock, cycloheximide (20 µM, final) was added to the culture. The culture was sampled and mixed with the luciferase substrate decanol (Sigma); and the signal was immediately measured in a luminometer (Luminoskan Ascent, Thermo Scientific).

### LacZ Reporter Gene Assay

DBY746 strains with either 4xSTRE- or 1xPDS-LacZ integrated in the *URA3* locus have been described previously [24]. One-day old cells grown in SDC were washed 3 times with water and resuspended in water (extreme CR/starvation). Four hours after the initiation of CR, glucose, glycerol, or ethanol (0.8%, final) were added to the cultures. Samples were collected after 2 and 4 hours of further incubation at 30°C with shaking. LacZ activity was measured as described previously [24].

## Supporting Information

Figure S1 Reduced Tor complex I (TORC1) activity enhances stress resistance. (A) Heat shock and oxidative stress resistance of wild type (DBY746) and cells deficient of either *TOR1* or TORC1 subunit *TCO89*. Day 3 cells were subject to heat stress (55°C for 100 min) or oxidative stress (H_2_O_2_, 100 mM for 60 min). (B) Overnight culture of wild type cells were diluted into fresh SDC medium (initial OD_600_ 0.3) and allowed to grow. Rapamycin was added to the culture after 5.5 hours (OD_600_ ∼1) or 9.5 hours (OD_600_ ∼6). Heat shock assay (55°C for 75 min) was performed at day 3. (C) Rapamycin was added to the SDC medium at the start of the culture with the concentrations indicated. Heat resistance assay (55°C for 75 min) was performed at day 3.
(0.14 MB PDF)

Figure S2 qRT-PCR validation of DNA microarray results. (A) Northern blot analysis of *YDL218W* and *SPS100* mRNA levels in wild type (DBY746), *tor1*Δ and *sch9*Δ at day 2.5 (equal amounts of total RNA were loaded). The PCR products of YDL218W (83 bp, see primer list below) and SPS100 (99 bp) labeled with random priming (Promega) were used as probes. (B-K) Quantitative RT-PCR analysis of mRNA levels in wild type (DBY746), *tor1*Δ, *sch9*Δ, *gis1*Δ, and *sch9*Δ *gis1*Δ mutants at day 2.5. Gene expression levels were normalized to actin (*ACT1*) and expressed as the percentage of wild type. 2–3 independent cultures were analyzed. * *p*<0.05, ** *p*<0.01, ANOVA, Tukeys' multiple comparison test, compared to wild type. Primers used for qRT-PCR: *ACT1,* F, 5′-AGCTCCAATGAACCCTAAATCA-3′; R, 5′-ACGACGTGAGTAACACCATCAC-3′; *ERG5,* F, 5′-TATTTGGTTACAGCAGCATTGG-3′; R, 5′-AACACAAACTGGCTTACCACCT-3′; *FMP45,* F, 5′-TCAATTTACCATCGTCGTTCAG-3′; R, 5′-AAAAATAGGGAAATCAGCAGCA-3′; *GPD1,* F, 5′-GGTTGGAAACATGTGGCTCT-3′; R, 5′-GGCAGGTTCTTCATTGGGTA-3′; *GPD2*, F, 5′-TTTCCCAGAATCCAAAGTCG-3′; R, 5′-CGGATTGACCGTTAAGCAAT-3′; *RHR2*, F, 5′-GTAAGCCTCACCCAGAACCA-3′; R, 5′-CAACGATTTTACAGCCAGCA-3′; *GRE1,* F, 5′-CCAAACTTACCGCGAAACTAAC-3′; R, 5′-GTAGCGGTTACTTTGAGCACCT-3′; *RIM15,* F, 5′-ACCTCTGCCAAAAATGGAACTA-3′; R, 5′-ATTGTATGAGCGATTCCGTTCT-3′; *SPS100,* F, 5′-ACTTTGGTTGCCGGTAGAGATA-3′; R, 5′-CCATTGAACATTCTTCTGACCA-3′; *TIS11,* F, 5′-TCAGAGAAGGAATCCTCAGCTC-3′; R, 5′-TTCGCACAGCTCTGTCTTGTAT-3′; *YDL218W,* F, 5′-AGGTATTTTGTGTCTGGCCCTA-3′; R, 5′-GCCATAGCATACAAACGATCAA-3′; *YLR012C,* F, 5′-CTTCAACTGCAACCTGAACAAC-3′; R, 5′-GATCGAACCAAGCAACTTCTTC-3′.
(0.06 MB PDF)

Figure S3 Chronological life span. (A) Life span of cells (DBY746 background) lacking the genes that were most up- (Fmp45, Ime1, Rpi1, and Ydl218w) or down-regulated (Ydr012c). Data represent mean and SEM of 2–5 experiments. (B–C) Chronological life span. Strains shown are wild type (DBY746), *ras2*Δ, *ras2*Δ *fmp45*Δ, *ras2*Δ *ydl218w*Δ, *tor1*Δ, and *tor1*Δ *fmp45*Δ. Data represent mean and SEM of 2–6 experiments. (D) Cells (BY4741 background) lacking key genes involved in ergosterol biosynthesis were subject to heat shock (55°C) or oxidative stress (H_2_O_2_, 150 mM for 60 min).
(0.20 MB PDF)

Figure S4 Glycerol metabolism in *tor1*Δ and *ras2*Δ mutants. (A) Glycerol concentration in the medium of wild type (DBY746), *tor1*Δ and *ras2*Δ cultures. Data represent the mean and SEM, n = 5. (B) Day 3 cells grown in SDC were washed 3 times with water and exposed to high concentrations of NaCl (2 M or 4 M) for 24 hours. The cells were then washed 3 times to remove the salt, serially diluted, and spotted on to YPD plate. (C) Yeast grown in SDC was sampled (1 ml) at indicated time points. [1,2,3-^3^H] Glycerol (ARC, Inc) was added to the aliquot and incubated at 30°C with shaking for 24 hours. Cells were then washed three times with water and resuspended in 2 ml scintillation fluid. The [^3^H]-content was determined by scintillation counting (Wallac 1410, Pharmacia) and normalized to the cell number (viability by CFU). Data represent the mean of two *tor1*Δ and *ras2*Δ cultures analyzed. Wild type, n = 4.
(0.05 MB PDF)

Figure S5 (A) Chronological life span. Strains shown are wild type (BY4741), *gpd1*Δ, and *gpd2*Δ. Data represent mean and SEM, n = 3. (B) Day 3 wild type (BY4741) and *sch9*Δ mutants expressing bacterial heat-sensitive luciferase were subject to heat stress (42°C for 60 min). Data represent mean and SEM, n = 3. (C) Recovery of luciferase activities after heat stress (42°C for 60 min) in wild type (BY4741) cells pre-treated with glycerol (with concentrations indicated) for 30 min. Data represent mean and SEM, n = 5.
(0.01 MB PDF)

Figure S6 Chronological life span. (A) Chronological life span of cells grown in reduced glucose medium (SC+0.5% glucose). Strains shown are wild type (DBY746) and *rhr2*Δ (in duplicate). (B) SDC cultures were switched to water at day3 (extreme CR/starvation). Strains shown are wild type and *rhr2*Δ (in duplicate). (C–D) *in situ* viability assay of *sch9*Δ (C, n = 3–5) and *ras2*Δ (D, n = 2) mutants in the presence of different carbon sources. Cells from day 1 SDC cultures were plated onto SC-Trp plates (no carbon source, CS), SC-Trp plates supplemented with 2% glucose (Glc), 3% glycerol (Gly), or agar plates (extreme CR/water).
(0.02 MB PDF)

Table S1 Chronological life span. Mean and maximum life span (10% survival) was calculated from curve fitting of the survival data (from pair matched, pooled experiments) with the statistical software Prism (GraphPad Software).* n, the number of cultures analyzed. ** *p*-value for mean CLS of mutants compared to that of wild type, ANOVA, Tukey's Multiple Comparison Test, except for *tor1*Δ *vs.* wild type, unpaired t-test, two-tailed.
(0.05 MB PDF)

Table S2 Microarray analysis of gene expression profiles at day 2.5. Data represent the log ratio of *sch9*Δ, *tor1*Δ, and *ras2*Δ to wild type (DBY746), n = 3.
(1.54 MB XLS)

Table S3 The significance of overlapping in up- and down-regulated genes between long-lived mutants based on hypergeometric distribution and Fisher's exact test.
(0.01 MB PDF)

Table S4 Gene ontology (GO) analysis of expression profiles of long-lived mutants. Significantly up-or down-regulated categories were shown (*p*<0.05). *q*-value was also calculated to correct the multi-testing error. *GO categories: C, Cellular component; F, molecular function; and P, biological process.
(0.04 MB PDF)

Table S5 Expression levels of genes involved in ribosomal structure (a and b), glycolysis-gluconeogenesis (c), glucose transport (d), TCA cycle (e), and oxidative phosphorylation (f) in long-lived mutants compared to wild type cells at day 2.5. Data were expressed in fold change, WT = 1. For illustration purpose, gene expression levels that are 20% higher or lower than wild type cells are marked in red or green, respectively.
(0.12 MB XLS)

Table S6 Expression levels of genes involved in glycerol metabolism in long-lived *sch9*Δ, *tor1*Δ, and *ras2*Δ mutants at day 2.5. The glycerol biosynthesis gene subset contains 21 genes. Denote the gene subset by S and the entire gene as a whole by G. We compared expressions of S against those in the complement of S in G denoted by G-S. The Wilcoxon rank test (one-sided) was performed. We found that glycerol biosynthesis gene set is up-regulated with *p*-value 0.0058 in *sch9*Δ, 0.0614 in *tor1*Δ, and 0.0142 in *ras2*Δ. For illustration purpose, gene expression levels that are 20% higher or lower than wild type cells are marked in red or green, respectively. ^1^ Data were expressed in fold change, WT = 1. ^2^ Array probe specificity. ^3^ Day 1.5-old SDC wild type (DBY746) cells were washed 3 times with water and incubated in water for 24 or 48 hours. Data represent fold change compared to wild type cells in SDC.
(0.02 MB XLS)

Table S7 Schematic representation of ergosterol biosynthesis and expression levels of genes involved in the process. Data were expressed in fold change, WT = 1. For illustration purpose, gene expression levels that are 20% higher or lower than wild type cells are marked in red or green, respectively.
(0.02 MB PDF)
