# Supplementary figures and images for: Tor1/Sch9-Regulated Carbon Source Substitution Is as Effective as Calorie Restriction in Life Span Extension
Source: PLoS Genet. 2009 May 8;5(5):e1000467. doi: 10.1371/journal.pgen.1000467 (PMC2669710; doi:10.1371/journal.pgen.1000467)

# Figure S1

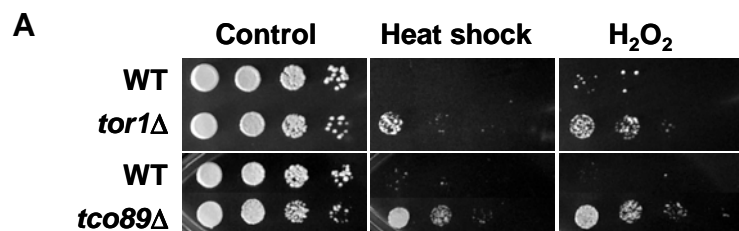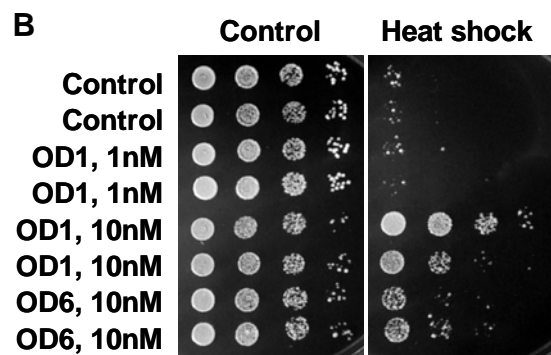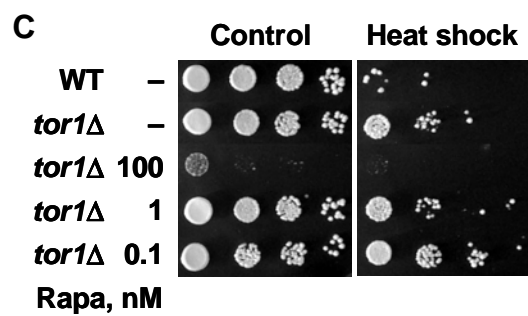

Supplement: Figure S1 — Reduced Tor complex I (TORC1) activity enhances stress resistance. (A) Heat shock and oxidative stress resistance of wild type (DBY746) and cells deficient of either TOR1 or TORC1 subunit TCO89. Day 3 cells were subject to heat stress (55°C for 100 min) or oxidative stress (H2O2, 100 mM for 60 min). (B) Overnight culture of wild type cells were diluted into fresh SDC medium (initial OD600 0.3) and allowed to grow. Rapamycin was added to the culture after 5.5 hours (OD600 ∼1) or 9.5 hours (OD600 ∼6). Heat shock assay (55°C for 75 min) was performed at day 3. (C) Rapamycin was added to the SDC medium at the start of the culture with the concentrations indicated. Heat resistance assay (55°C for 75 min) was performed at day 3. (0.14 MB PDF) [file pgen.1000467.s001.pdf]

# Figure S2

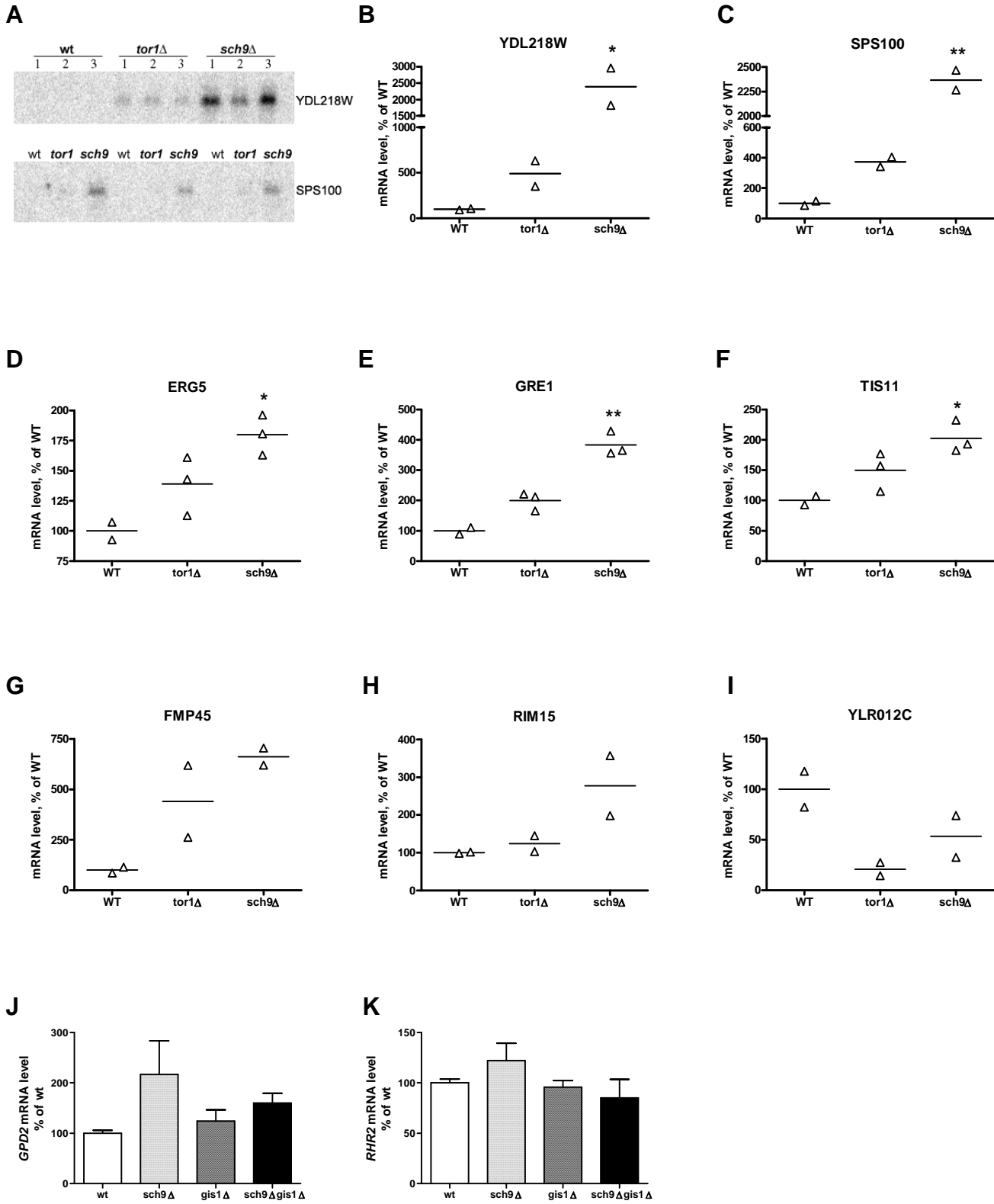

Supplement: Figure S2 — qRT-PCR validation of DNA microarray results. (A) Northern blot analysis of YDL218W and SPS100 mRNA levels in wild type (DBY746), tor1Δ and sch9Δ at day 2.5 (equal amounts of total RNA were loaded). The PCR products of YDL218W (83 bp, see primer list below) and SPS100 (99 bp) labeled with random priming (Promega) were used as probes. (B-K) Quantitative RT-PCR analysis of mRNA levels in wild type (DBY746), tor1Δ, sch9Δ, gis1Δ, and sch9Δ gis1Δ mutants at day 2.5. Gene expression levels were normalized to actin (ACT1) and expressed as the percentage of wild type. 2–3 independent cultures were analyzed. * p<0.05, ** p<0.01, ANOVA, Tukeys' multiple comparison test, compared to wild type. Primers used for qRT-PCR: ACT1, F, 5′-AGCTCCAATGAACCCTAAATCA-3′; R, 5′-ACGACGTGAGTAACACCATCAC-3′; ERG5, F, 5′-TATTTGGTTACAGCAGCATTGG-3′; R, 5′-AACACAAACTGGCTTACCACCT-3′; FMP45, F, 5′-TCAATTTACCATCGTCGTTCAG-3′; R, 5′-AAAAATAGGGAAATCAGCAGCA-3′; GPD1, F, 5′-GGTTGGAAACATGTGGCTCT-3′; R, 5′-GGCAGGTTCTTCATTGGGTA-3′; GPD2, F, 5′-TTTCCCAGAATCCAAAGTCG-3′; R, 5′-CGGATTGACCGTTAAGCAAT-3′; RHR2, F, 5′-GTAAGCCTCACCCAGAACCA-3′; R, 5′-CAACGATTTTACAGCCAGCA-3′; GRE1, F, 5′-CCAAACTTACCGCGAAACTAAC-3′; R, 5′-GTAGCGGTTACTTTGAGCACCT-3′; RIM15, F, 5′-ACCTCTGCCAAAAATGGAACTA-3′; R, 5′-ATTGTATGAGCGATTCCGTTCT-3′; SPS100, F, 5′-ACTTTGGTTGCCGGTAGAGATA-3′; R, 5′-CCATTGAACATTCTTCTGACCA-3′; TIS11, F, 5′-TCAGAGAAGGAATCCTCAGCTC-3′; R, 5′-TTCGCACAGCTCTGTCTTGTAT-3′; YDL218W, F, 5′-AGGTATTTTGTGTCTGGCCCTA-3′; R, 5′-GCCATAGCATACAAACGATCAA-3′; YLR012C, F, 5′-CTTCAACTGCAACCTGAACAAC-3′; R, 5′-GATCGAACCAAGCAACTTCTTC-3′. (0.06 MB PDF) [file pgen.1000467.s002.pdf]

# Figure S3

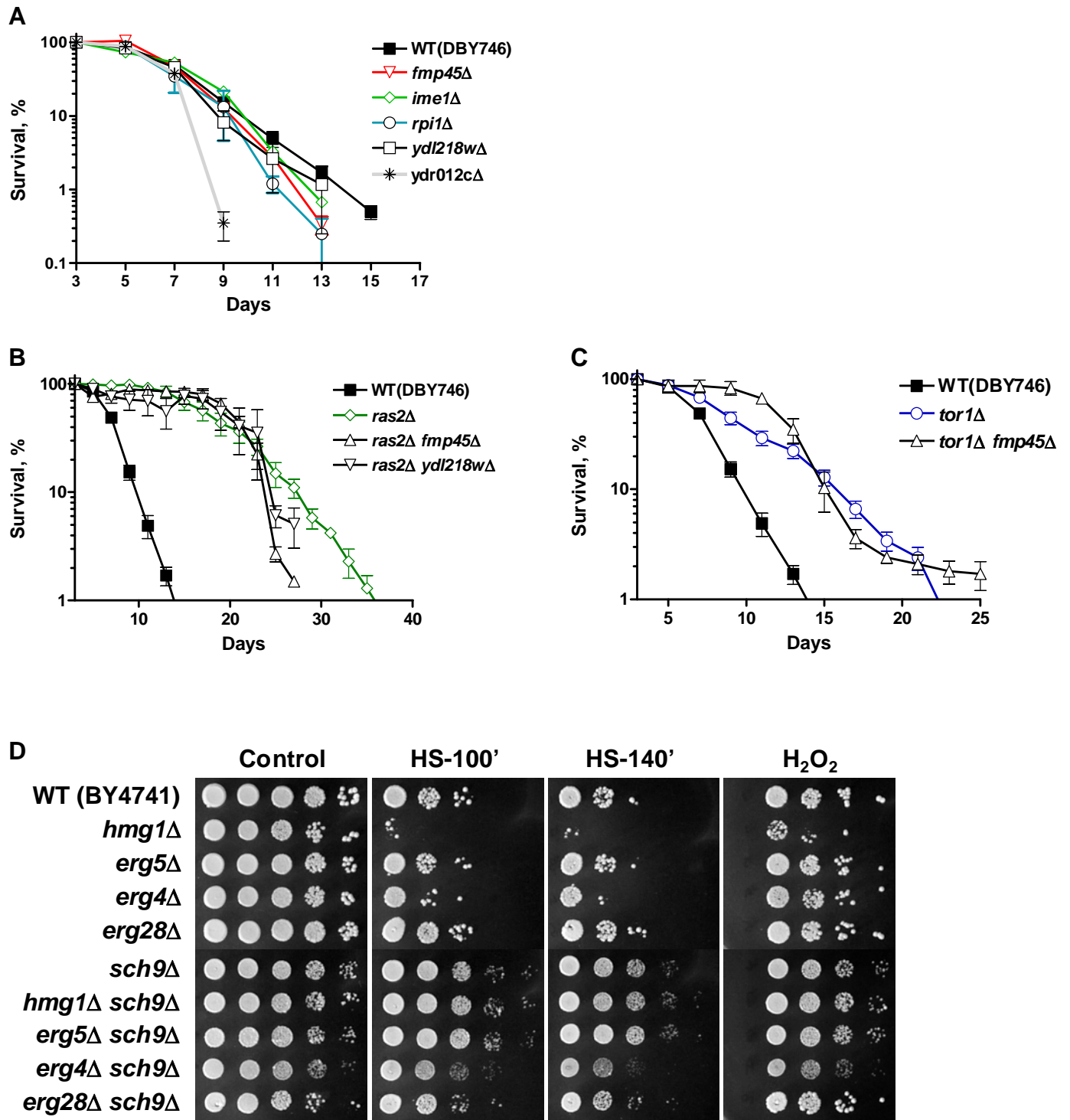

Supplement: Figure S3 — Chronological life span. (A) Life span of cells (DBY746 background) lacking the genes that were most up- (Fmp45, Ime1, Rpi1, and Ydl218w) or down-regulated (Ydr012c). Data represent mean and SEM of 2–5 experiments. (B–C) Chronological life span. Strains shown are wild type (DBY746), ras2Δ, ras2Δ fmp45Δ, ras2Δ ydl218wΔ, tor1Δ, and tor1Δ fmp45Δ. Data represent mean and SEM of 2–6 experiments. (D) Cells (BY4741 background) lacking key genes involved in ergosterol biosynthesis were subject to heat shock (55°C) or oxidative stress (H2O2, 150 mM for 60 min). (0.20 MB PDF) [file pgen.1000467.s003.pdf]

Figure S4

A

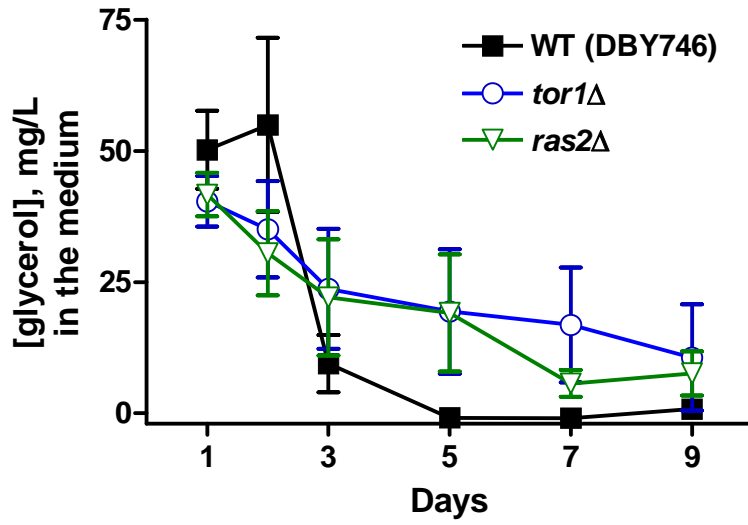

B

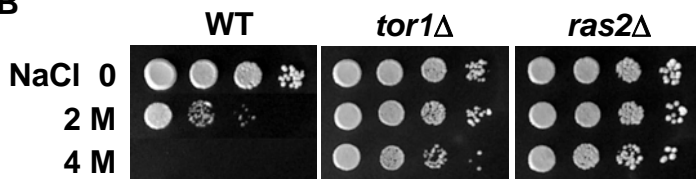

C

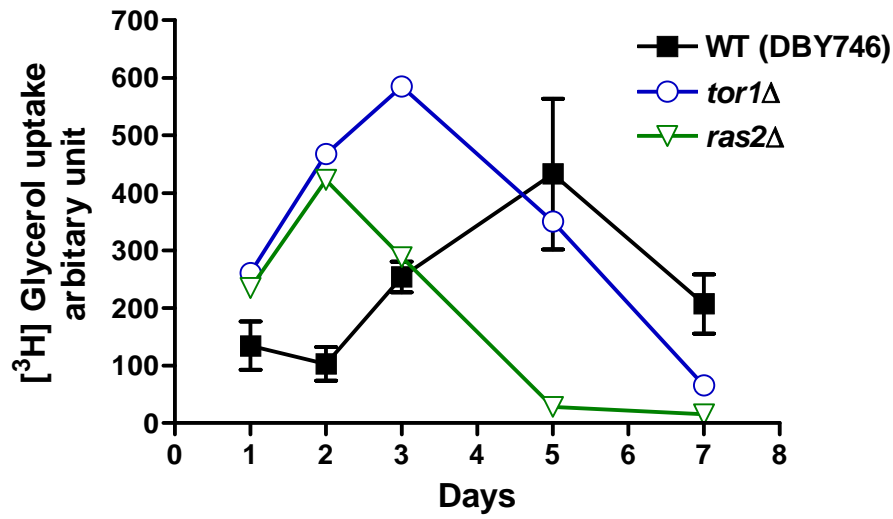

Supplement: Figure S4 — Glycerol metabolism in tor1Δ and ras2Δ mutants. (A) Glycerol concentration in the medium of wild type (DBY746), tor1Δ and ras2Δ cultures. Data represent the mean and SEM, n = 5. (B) Day 3 cells grown in SDC were washed 3 times with water and exposed to high concentrations of NaCl (2 M or 4 M) for 24 hours. The cells were then washed 3 times to remove the salt, serially diluted, and spotted on to YPD plate. (C) Yeast grown in SDC was sampled (1 ml) at indicated time points. [1,2,3-3H] Glycerol (ARC, Inc) was added to the aliquot and incubated at 30°C with shaking for 24 hours. Cells were then washed three times with water and resuspended in 2 ml scintillation fluid. The [3H]-content was determined by scintillation counting (Wallac 1410, Pharmacia) and normalized to the cell number (viability by CFU). Data represent the mean of two tor1Δ and ras2Δ cultures analyzed. Wild type, n = 4. (0.05 MB PDF) [file pgen.1000467.s004.pdf]

# Figure S5

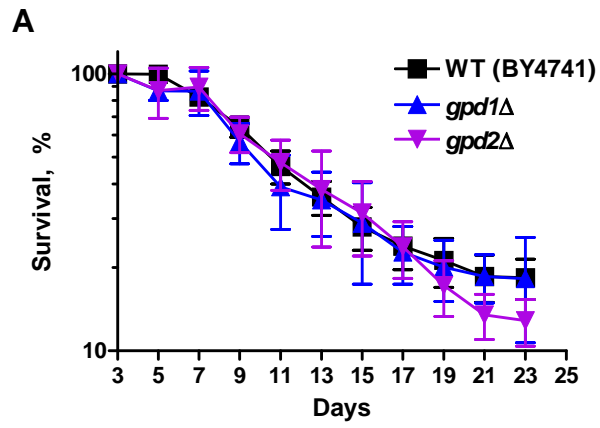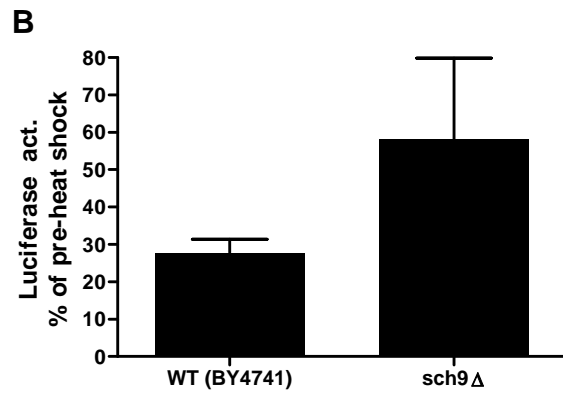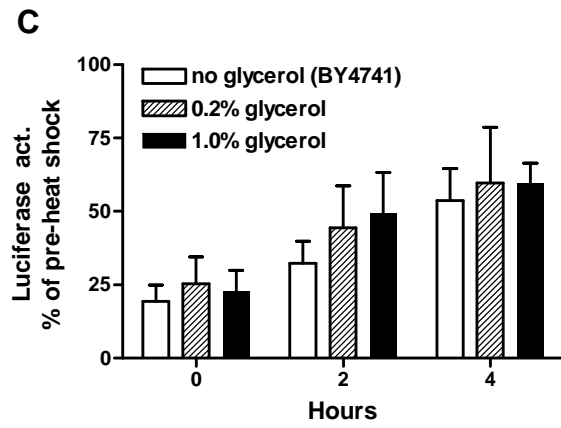

Supplement: Figure S5 — (A) Chronological life span. Strains shown are wild type (BY4741), gpd1Δ, and gpd2Δ. Data represent mean and SEM, n = 3. (B) Day 3 wild type (BY4741) and sch9Δ mutants expressing bacterial heat-sensitive luciferase were subject to heat stress (42°C for 60 min). Data represent mean and SEM, n = 3. (C) Recovery of luciferase activities after heat stress (42°C for 60 min) in wild type (BY4741) cells pre-treated with glycerol (with concentrations indicated) for 30 min. Data represent mean and SEM, n = 5. (0.01 MB PDF) [file pgen.1000467.s005.pdf]

# Figure S6

**A**

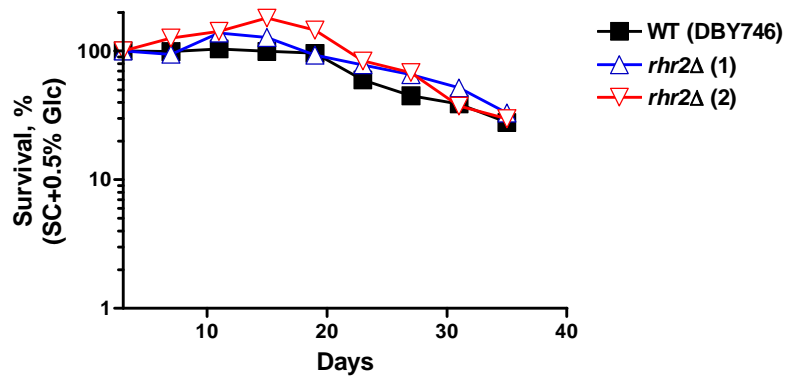

**B**

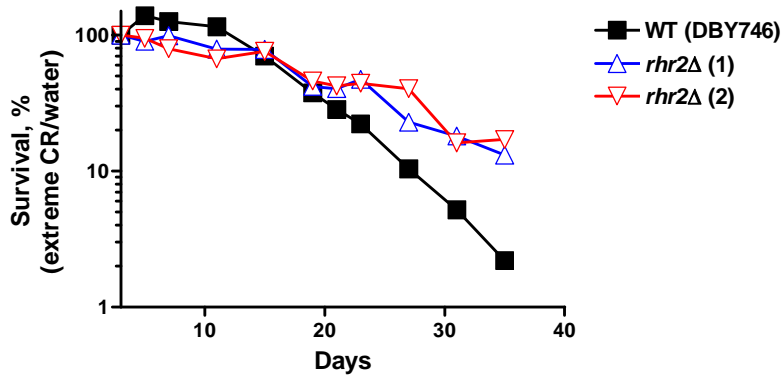

**C**

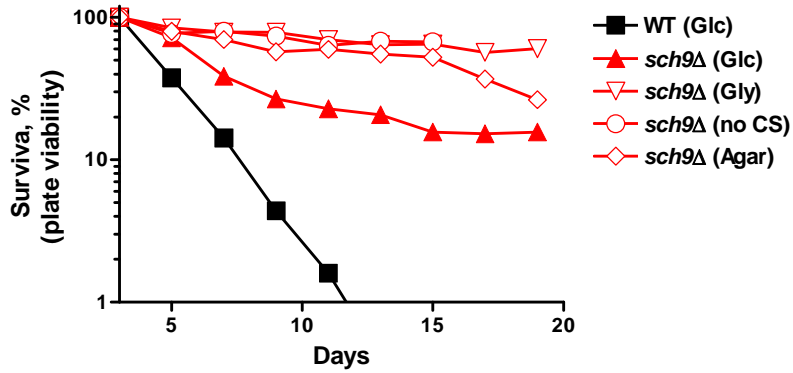

**D**

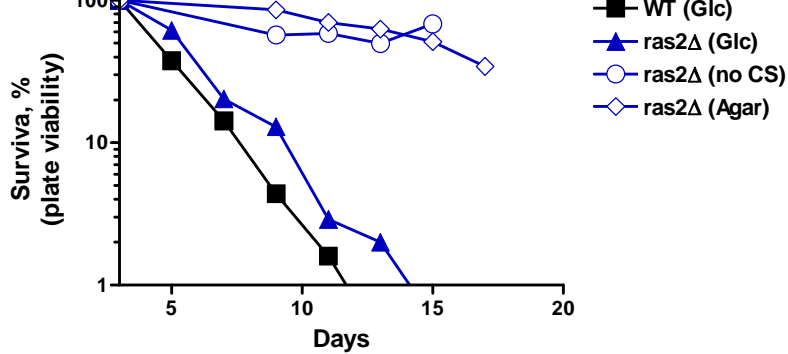

Supplement: Figure S6 — Chronological life span. (A) Chronological life span of cells grown in reduced glucose medium (SC+0.5% glucose). Strains shown are wild type (DBY746) and rhr2Δ (in duplicate). (B) SDC cultures were switched to water at day3 (extreme CR/starvation). Strains shown are wild type and rhr2Δ (in duplicate). (C–D) in situ viability assay of sch9Δ (C, n = 3–5) and ras2Δ (D, n = 2) mutants in the presence of different carbon sources. Cells from day 1 SDC cultures were plated onto SC-Trp plates (no carbon source, CS), SC-Trp plates supplemented with 2% glucose (Glc), 3% glycerol (Gly), or agar plates (extreme CR/water). (0.02 MB PDF) [file pgen.1000467.s006.pdf]
