# Supplementary material for: Tor1/Sch9-Regulated Carbon Source Substitution Is as Effective as Calorie Restriction in Life Span Extension
Source: PLoS Genet. 2009 May 8;5(5):e1000467. doi: 10.1371/journal.pgen.1000467 (PMC2669710; doi:10.1371/journal.pgen.1000467)
Supplement: Table S1 — Chronological life span. Mean and maximum life span (10% survival) was calculated from curve fitting of the survival data (from pair matched, pooled experiments) with the statistical software Prism (GraphPad Software).* n, the number of cultures analyzed. ** p-value for mean CLS of mutants compared to that of wild type, ANOVA, Tukey's Multiple Comparison Test, except for tor1Δ vs. wild type, unpaired t-test, two-tailed. (0.05 MB PDF) [file pgen.1000467.s007.pdf]

Table S1. Chronological life span of various mutants.

	Mean CLS		10% survival			
	Days	% of WT	Days	% of WT	n*	*p***
WT (DBY746)	6.5	100	10.8	100	48	
*tor1*Δ	8.4	129	16.3	151	28	$p<0.001$**
*tco89*Δ	7.5	115	-	-	5	
*sch9*Δ	15.3	235	27.3	253	31	$p<0.001$
*ras2*Δ	18.3	282	29.2	270	14	$p<0.001$
*ras2*Δ *tor1*Δ	15.7	242	28.5	264	4	
*ras2*Δ *sch9*Δ	34.3	528	-	-	8	$p<0.001$
*sch9*Δ *tor1*Δ	15.0	231	28.1	260	6	$p<0.001$
*ras2*Δ *sch9*Δ *tor1*Δ	32.4	498	-	-	5	$p<0.001$
$RAS2^{Val19}$	7.3	112	10.0	93	4	
SCH9	6.8	105	9.8	91	8	
*tor1*Δ $RAS2^{Val19}$	6.6	102	10.5	97	4	
*tor1*Δ *SCH9*	6.3	97	9.5	88	8	
*sch9*Δ *ydl218w*Δ	11.4	176	23.7	220	8	
*sch9*Δ *ime1*Δ	18.9	291	28.0	259	3	
*sch9*Δ *rpi1*Δ	16.2	249	26.3	244	5	
*sch9*Δ *fmp45*Δ	11.8	182	21.9	203	2	
