# Supplementary material for: Tor1/Sch9-Regulated Carbon Source Substitution Is as Effective as Calorie Restriction in Life Span Extension
Source: PLoS Genet. 2009 May 8;5(5):e1000467. doi: 10.1371/journal.pgen.1000467 (PMC2669710; doi:10.1371/journal.pgen.1000467)
Supplement: Table S3 — The significance of overlapping in up- and down-regulated genes between long-lived mutants based on hypergeometric distribution and Fisher's exact test. (0.01 MB PDF) [file pgen.1000467.s009.pdf]

Table S3. The significance of overlapping in up- and down-regulated genes between long-lived mutants.

		p
*tor1*Δ *vs.sch9*Δ		
up	sum(dhyper(76:130,145, 5667-145,130))	3.31e-95
down	sum(dhyper(30:61,121, 5667-121,61))	2.31e-35
*ras2*Δ *vs.sch9*Δ		
up	sum(dhyper(98:322,145, 5667-145,322))	6.54e-92
down	sum(dhyper(72:349,118, 5667-118,349))	5.68e-59
*tor1*Δ *vs.ras2*Δ		
up	sum(dhyper(94:130,322, 5667-322,130))	1.77e-92
down	sum(dhyper(43:61,349, 5667-349,61))	3.43e-39
