# Supplementary material for: Tor1/Sch9-Regulated Carbon Source Substitution Is as Effective as Calorie Restriction in Life Span Extension
Source: PLoS Genet. 2009 May 8;5(5):e1000467. doi: 10.1371/journal.pgen.1000467 (PMC2669710; doi:10.1371/journal.pgen.1000467)
Supplement: Table S4 — Gene ontology (GO) analysis of expression profiles of long-lived mutants. Significantly up-or down-regulated categories were shown (p<0.05). q-value was also calculated to correct the multi-testing error. *GO categories: C, Cellular component; F, molecular function; and P, biological process. (0.04 MB PDF) [file pgen.1000467.s010.pdf]

Table S4 Gene ontology (GO) analysis of expression profiles of long-lived mutants.

Positively affected TIGO categories

GO*	GO ID	Gene #	Annotation	*sch9*Δ p-value	*sch9*Δ q-value	*tor1*Δ p-value	*tor1*Δ q-value	*ras2*Δ p-value	*ras2*Δ q-value
C	GO:0005842	93	cytosolic large ribosomal subunit	**0**	**0**	**0**	**0**	**1.64E-12**	**2.37E-10**
C	GO:0005843	63	cytosolic small ribosomal subunit	**0**	**0**	**0**	**0**	**7.49E-09**	**6.49E-07**
F	GO:0003735	229	structural constituent of ribosome	**1.82E-05**	**6.86E-04**	**1.41E-05**	**5.57E-04**	5.45E-01	8.71E-01
P	GO:0007047	140	cell wall organization and biogenesis	**2.35E-04**	**5.51E-03**	**3.52E-02**	1.99E-01	**4.68E-03**	5.38E-02
C	GO:0005794	130	Golgi apparatus	**4.34E-04**	**9.18E-03**	**2.97E-02**	1.81E-01	3.33E-01	8.71E-01
P	GO:0042364	36	water-soluble vitamin biosynthesis	**8.72E-04**	**1.51E-02**	**1.27E-02**	1.04E-01	**2.25E-04**	**5.51E-03**
P	GO:0046365	33	monosaccharide catabolism	**1.32E-03**	**2.01E-02**	**2.94E-05**	**1.02E-03**	**8.81E-06**	**3.81E-04**
P	GO:0016125	37	sterol metabolism	**5.65E-03**	6.20E-02	**7.50E-03**	7.56E-02	**7.51E-05**	**2.32E-03**
P	GO:0006487	43	protein amino acid N-linked glycosylation	**6.94E-03**	7.16E-02	**3.10E-02**	1.85E-01	**4.99E-05**	**1.66E-03**
C	GO:0031226	31	intrinsic to plasma membrane	**8.60E-03**	8.19E-02	**2.91E-02**	1.80E-01	**7.05E-05**	**2.26E-03**
F	GO:0000030	42	mannosyltransferase activity	**1.06E-02**	9.44E-02	6.23E-02	2.83E-01	**9.70E-05**	**2.90E-03**
F	GO:0046873	38	metal ion transporter activity	**1.38E-02**	1.09E-01	**2.32E-02**	1.55E-01	**1.39E-04**	**3.77E-03**
F	GO:0015082	35	di-, tri-valent inorganic cation transporter activity	**2.27E-02**	1.55E-01	**1.25E-02**	1.03E-01	**1.20E-04**	**3.48E-03**
C	GO:0005789	115	endoplasmic reticulum membrane	**2.36E-02**	1.56E-01	**5.51E-03**	6.12E-02	**6.46E-07**	**4.59E-05**
C	GO:0005811	33	lipid particle	5.19E-02	2.56E-01	6.89E-02	3.02E-01	5.16E-02	2.55E-01
P	GO:0008643	32	carbohydrate transport	1.45E-01	5.24E-01	**4.72E-03**	5.38E-02	**1.89E-06**	**1.09E-04**
P	GO:0030384	31	phosphoinositide metabolism	5.64E-01	8.71E-01	5.82E-01	8.71E-01	**4.60E-04**	**9.49E-03**
C	GO:0009277	111	cell wall	8.91E-01	8.71E-01	**6.19E-06**	**2.98E-04**	**5.65E-06**	**2.88E-04**
P	GO:0046474	34	glycerophospholipid biosynthesis	9.85E-01	8.71E-01	8.64E-01	8.71E-01	**4.19E-02**	2.21E-01

Negatively affected TIGO categories

GO*	GO ID	Gene #	Annotation	*sch9*Δ p-value	*sch9*Δ q-value	*tor1*Δ p-value	*tor1*Δ q-value	*ras2*Δ p-value	*ras2*Δ q-value
C	GO:0005762	43	mitochondrial large ribosomal subunit	**1.56E-19**	**3.32E-17**	**1.13E-20**	**4.29E-18**	**1.34E-20**	**4.29E-18**
C	GO:0005763	34	mitochondrial small ribosomal subunit	**6.94E-13**	**4.93E-11**	**3.17E-13**	**2.54E-11**	**4.83E-14**	**4.41E-12**
C	GO:0016591	74	DNA-directed RNA polymerase II, holoenzyme	**1.61E-05**	**2.29E-04**	**9.05E-05**	**8.65E-04**	**4.97E-10**	**2.27E-08**
C	GO:0000778	47	condensed nuclear chromosome kinetochore	**2.24E-02**	5.37E-02	8.02E-02	1.34E-01	**4.68E-04**	**2.88E-03**
C	GO:0005875	35	microtubule associated complex	**4.31E-02**	8.61E-02	3.67E-01	4.20E-01	**2.50E-03**	**1.12E-02**
C	GO:0016585	71	chromatin remodeling complex	6.61E-02	1.16E-01	**3.38E-03**	**1.39E-02**	**9.96E-08**	**3.04E-06**
C	GO:0000502	46	proteasome complex	**3.92E-04**	**2.56E-03**	**4.51E-03**	**1.72E-02**	**1.35E-08**	**4.79E-07**
C	GO:0005684	37	major (U2-dependent) spliceosome	**2.27E-02**	5.40E-02	1.45E-01	2.08E-01	**7.28E-05**	**7.39E-04**
C	GO:0000123	39	histone acetyltransferase complex	**1.87E-02**	**4.77E-02**	**3.39E-03**	**1.39E-02**	**1.52E-05**	**2.21E-04**
C	GO:0046540	30	U4/U6 x U5 tri-snRNP complex	1.82E-01	2.48E-01	6.38E-01	6.11E-01	**1.44E-02**	**4.05E-02**
C	GO:0030880	32	RNA polymerase complex	**6.80E-03**	**2.30E-02**	**1.65E-02**	**4.38E-02**	5.73E-02	1.05E-01
C	GO:0000131	35	incipient bud site	6.35E-01	6.09E-01	**4.38E-02**	8.72E-02	**2.22E-03**	**1.02E-02**
C	GO:0005934	50	bud tip	4.39E-01	4.73E-01	1.87E-01	2.55E-01	**2.13E-03**	**9.94E-03**
C	GO:0000790	36	nuclear chromatin	3.85E-01	4.30E-01	1.64E-01	2.29E-01	**1.76E-03**	**8.53E-03**
C	GO:0005643	50	nuclear pore	8.66E-02	1.42E-01	**8.60E-03**	**2.78E-02**	**1.43E-03**	**7.34E-03**
C	GO:0005743	158	mitochondrial inner membrane	**2.64E-16**	**2.82E-14**	**3.56E-17**	**5.70E-15**	**3.14E-09**	**1.34E-07**
F	GO:0003924	59	GTPase activity	**3.73E-02**	7.82E-02	6.45E-02	1.13E-01	**3.66E-05**	**4.59E-04**

F	GO:0016251	62	general RNA polymerase II transcription factor activity	**1.87E-02**	**4.77E-02**	**2.18E-02**	5.30E-02	**1.38E-06**	**3.04E-05**
F	GO:0005478	30	intracellular transporter activity	1.07E-01	1.65E-01	2.46E-01	3.18E-01	**1.20E-03**	**6.41E-03**
F	GO:0005083	58	small GTPase regulator activity	9.81E-01	6.43E-01	2.93E-01	3.61E-01	**1.87E-02**	**4.77E-02**
F	GO:0008080	37	N-acetyltransferase activity	**6.89E-03**	**2.32E-02**	**6.43E-03**	**2.25E-02**	**3.16E-04**	**2.20E-03**
F	GO:0003899	33	DNA-directed RNA polymerase activity	**4.52E-03**	**1.72E-02**	**9.58E-03**	**3.02E-02**	**4.26E-02**	8.54E-02
F	GO:0008757	61	S-adenosylmethionine-dependent methyltransferase activity	**3.68E-03**	**1.48E-02**	**5.29E-03**	**1.98E-02**	1.16E-01	1.76E-01
F	GO:0004519	56	endonuclease activity	5.26E-02	9.84E-02	**4.90E-02**	9.28E-02	**1.71E-03**	**8.41E-03**
F	GO:0004540	100	ribonuclease activity	5.73E-02	1.05E-01	**3.00E-03**	**1.30E-02**	**4.49E-04**	**2.79E-03**
F	GO:0004527	33	exonuclease activity	1.00E-01	1.58E-01	**1.84E-03**	**8.87E-03**	**1.21E-03**	**6.41E-03**
F	GO:0003724	41	RNA helicase activity	**3.01E-02**	6.75E-02	**4.44E-04**	**2.79E-03**	**4.07E-02**	8.30E-02
F	GO:0008026	37	ATP-dependent helicase activity	6.70E-02	1.17E-01	**1.41E-03**	**7.30E-03**	**3.22E-02**	7.10E-02
F	GO:0008565	50	protein transporter activity	**2.70E-04**	**1.94E-03**	**7.99E-05**	**7.87E-04**	**1.48E-02**	**4.11E-02**
P	GO:0008652	99	amino acid biosynthesis	6.61E-01	6.22E-01	**2.00E-06**	**4.13E-05**	4.07E-01	4.48E-01
P	GO:0000398	97	nuclear mRNA splicing, via spliceosome	**1.01E-02**	**3.14E-02**	6.60E-02	1.16E-01	**6.16E-06**	**1.01E-04**
P	GO:0006906	30	vesicle fusion	1.89E-01	2.57E-01	3.05E-01	3.68E-01	**5.34E-03**	**1.99E-02**
P	GO:0006367	44	transcription initiation from RNA polymerase II promoter	**3.23E-02**	7.10E-02	**8.55E-03**	**2.78E-02**	**9.23E-06**	**1.37E-04**
P	GO:0006406	60	mRNA-nucleus export	**4.54E-02**	8.79E-02	**3.70E-04**	**2.44E-03**	**1.37E-04**	**1.10E-03**
P	GO:0001403	30	invasive growth	5.40E-01	5.41E-01	**1.61E-02**	**4.32E-02**	**2.17E-03**	**1.00E-02**
P	GO:0016570	59	histone modification	**1.56E-03**	**7.85E-03**	**2.16E-04**	**1.64E-03**	**7.30E-06**	**1.14E-04**
P	GO:0006473	38	protein amino acid acetylation	**1.15E-02**	**3.45E-02**	**6.17E-03**	**2.19E-02**	**1.16E-04**	**1.00E-03**
P	GO:0006365	67	35S primary transcript processing	**1.93E-03**	**9.16E-03**	**3.84E-06**	**7.23E-05**	**4.05E-03**	**1.59E-02**
P	GO:0006383	38	transcription from RNA polymerase III promoter	**2.18E-02**	5.30E-02	**2.40E-02**	5.66E-02	**6.19E-03**	**2.19E-02**
P	GO:0043414	35	biopolymer methylation	**1.09E-03**	**5.93E-03**	**2.94E-03**	**1.28E-02**	**1.30E-02**	**3.78E-02**
P	GO:0030490	46	processing of 20S pre-rRNA	**3.45E-04**	**2.33E-03**	**1.32E-06**	**3.01E-05**	**4.18E-02**	8.44E-02
P	GO:0007005	95	mitochondrion organization and biogenesis	**6.62E-05**	**7.02E-04**	**1.32E-04**	**1.07E-03**	**4.51E-06**	**8.02E-05**
P	GO:0006402	55	mRNA catabolism	6.97E-02	1.21E-01	**3.06E-03**	**1.30E-02**	**9.57E-04**	**5.33E-03**
P	GO:0006289	31	nucleotide-excision repair	1.18E-01	1.77E-01	**3.42E-02**	7.36E-02	**1.74E-03**	**8.48E-03**
P	GO:0045944	48	positive regulation of transcription from RNA polymerase II promoter	1.10E-01	1.67E-01	**1.63E-04**	**1.27E-03**	**4.17E-05**	**5.10E-04**
P	GO:0000154	89	rRNA modification	**4.95E-02**	9.35E-02	**1.09E-03**	**5.93E-03**	1.89E-01	2.57E-01
P	GO:0006611	44	protein-nucleus export	**4.06E-02**	8.30E-02	**6.21E-04**	**3.71E-03**	**6.76E-03**	**2.30E-02**
P	GO:0006413	46	translational initiation	**2.03E-02**	5.08E-02	**1.07E-03**	**5.91E-03**	**3.58E-02**	7.63E-02
P	GO:0016044	31	membrane organization and biogenesis	**2.09E-03**	**9.85E-03**	**1.38E-03**	**7.18E-03**	**9.74E-03**	**3.06E-02**
P	GO:0009064	43	glutamine family amino acid metabolism	**4.85E-02**	9.24E-02	**8.42E-05**	**8.16E-04**	1.26E-01	1.86E-01
P	GO:0006626	47	protein-mitochondrial targeting	**8.33E-06**	**1.27E-04**	**1.46E-06**	**3.11E-05**	**4.04E-04**	**2.59E-03**
P	GO:0009060	82	aerobic respiration	**2.66E-08**	**8.96E-07**	**4.73E-09**	**1.78E-07**	**1.32E-06**	**3.01E-05**
P	GO:0006119	46	oxidative phosphorylation	**7.03E-07**	**2.04E-05**	**9.01E-07**	**2.31E-05**	**1.57E-04**	**1.24E-03**
P	GO:0006118	31	electron transport	**1.22E-04**	**1.03E-03**	**1.01E-04**	**9.28E-04**	**4.29E-03**	**1.65E-02**
