# Supplementary material for: Tor1/Sch9-Regulated Carbon Source Substitution Is as Effective as Calorie Restriction in Life Span Extension
Source: PLoS Genet. 2009 May 8;5(5):e1000467. doi: 10.1371/journal.pgen.1000467 (PMC2669710; doi:10.1371/journal.pgen.1000467)
Supplement: Table S7 — Schematic representation of ergosterol biosynthesis and expression levels of genes involved in the process. Data were expressed in fold change, WT = 1. For illustration purpose, gene expression levels that are 20% higher or lower than wild type cells are marked in red or green, respectively. (0.02 MB PDF) [file pgen.1000467.s013.pdf]

Table S7. Expression levels of genes involved in ergosterol biosynthesis

Adapted from SGD

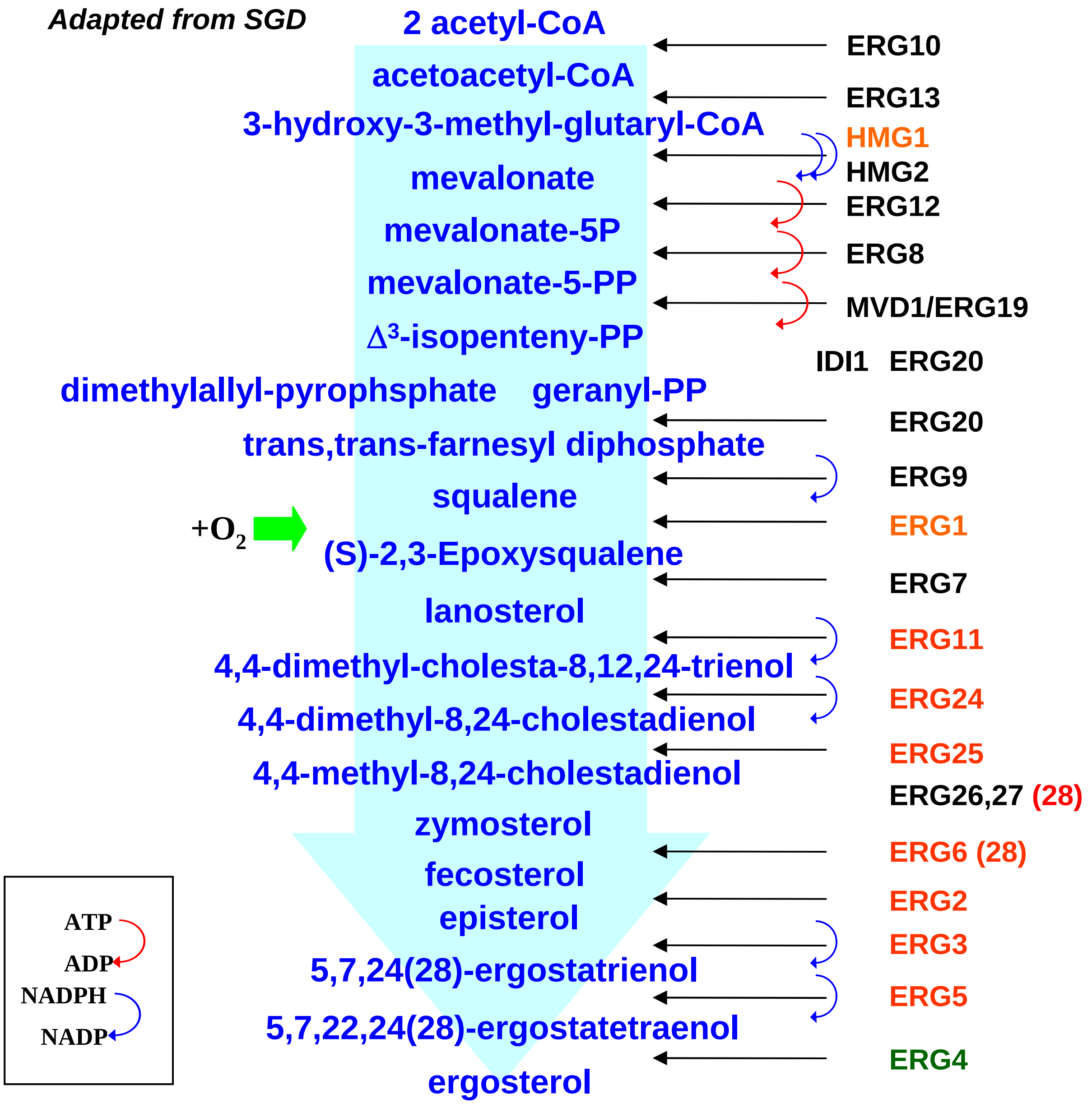

Public ID	Gene	*sch9Δ*	*tor1Δ*	*ras2Δ*
YPL028W	*ERG10*	1.00	1.12	1.18
YML126C	*ERG13*	1.18	1.17	1.26
YML075C	*HMG1*	1.47	1.65	1.59
YLR450W	*HMG2*	1.01	1.11	1.41
YMR208W	*ERG12*	1.23	1.12	1.53
YMR220W	*ERG8*	1.17	1.07	0.75
YNR043W	*MVD1*	0.62	0.89	1.22
YPL117C	*IDI1*	0.87	0.84	0.61
YJL167W	*ERG20*	1.01	1.19	0.80
YHR190W	*ERG9*	0.96	0.97	0.93
YGR175C	*ERG1*	1.31	1.26	1.39
YHR072W	*ERG7*	0.84	0.69	1.00
YHR007C	*ERG11*	1.48	1.38	1.38
YNL280C	*ERG24*	1.53	1.85	1.86
YGR060W	*ERG25*	2.33	2.59	2.02
YML008C	*ERG6*	1.77	2.23	1.15
YMR202W	*ERG2*	1.58	1.95	1.93
YLR056W	*ERG3*	1.83	1.99	1.61
YMR015C	*ERG5*	4.85	4.35	5.28
YGL012W	*ERG4*	0.63	0.78	0.80
YGL001C	*ERG26*	0.75	0.99	1.15
YLR100W	*ERG27*	1.24	1.23	1.07
YER044C	*ERG28*	2.02	1.70	2.50
